# Oxidative Neutralisation of Sulfur‐Based Chemical Warfare Agents Mediated by a Lipase: From Batch to Flow Reactor

**DOI:** 10.1002/chem.202403701

**Published:** 2025-03-12

**Authors:** Maxime Boddaert, Valmir Baptista da Silva, Sergui Mansour, Daniela Vuluga, Pierre‐Yves Renard, Jean‐Christophe M. Monbaliu, Julien Legros

**Affiliations:** ^1^ Univ Rouen Normandie INSA Rouen Normandie CNRS Normandie Univ CARMeN UMR 6064 F-76000 Rouen France; ^2^ Center for Integrated Technology and Organic Synthesis MolSys Research Unit University of Liège B-4000 Liège (Sart Tilman) Belgium; ^3^ INSA Rouen Normandie Univ Rouen Normandie CNRS PBS 76000 Rouen France; ^4^ WEL Research Institute Avenue Pasteur 6 B-1300 Wavre Belgium

**Keywords:** Chemical weapons, Yperite, VX, Biocatalysis, Flow chemistry

## Abstract

The sulfur‐containing chemical warfare agents sulfur mustard HD and nerve agent VX are highly toxic and persistent in the environment. Therefore, their neutralisation requires harsh oxidation conditions, but also precise selectivity. Here we report the safe and effective detoxification of surrogates CEES and PhX by selective oxidation of the sulfur atom by generating peracetic acid from AcOEt and aq. H_2_O_2_ assisted by the supported lipase CALB. Morever, it is possible to perform these neutralisations with safe ‘on demand’ generation of AcOOH in a flow system by using a packed bed reactor containing the supported biocatalyst.

## Graphical abstract

The simulants of the toxic and persistent blister agent sulfur mustard (CEES) and organophosphorous nerve agent VX (PhX) are neutralized with high selectivity by peracetic acid under batch and flow conditions. The hazardous peracetic acid is safely generated ‘on demand’ from AcOEt and aq. H_2_O_2_, assisted by the supported lipase CALB.

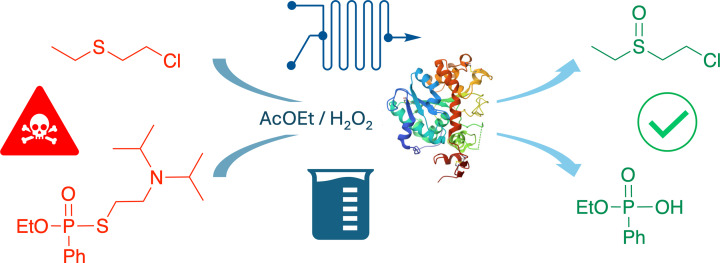

## Introduction

**Figure 1:**
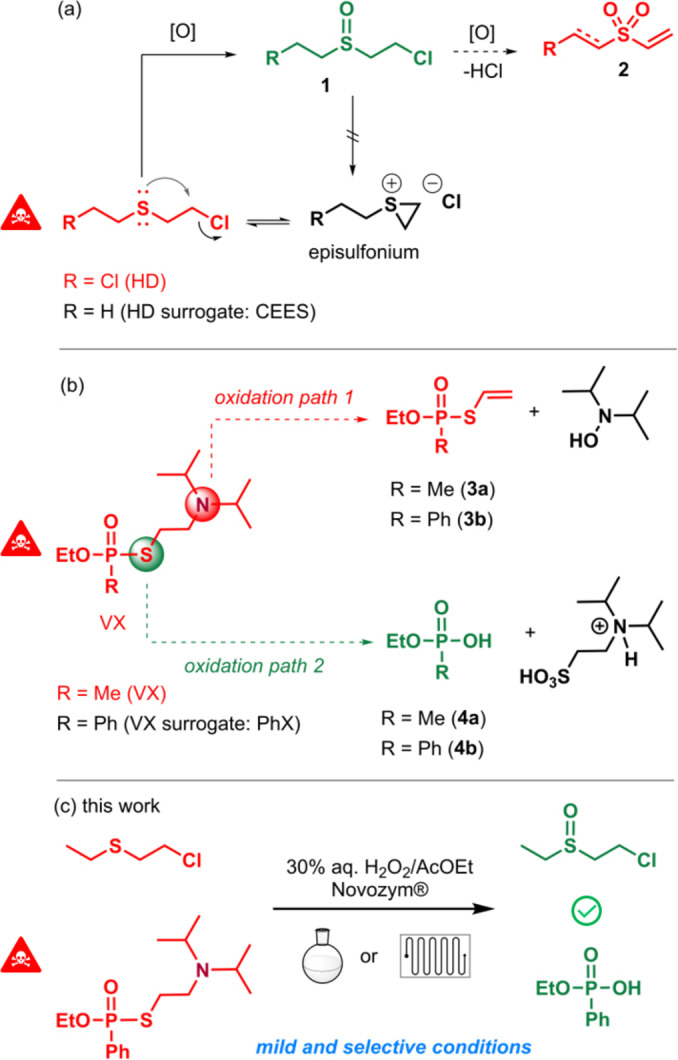
Oxidative paths of (a) sulfur mustard HD (surrogate: CEES) and (b) V‐series nerve agent VX (surrogate: PhX) through sulfur oxidation. (c) This work.

Chemical warfare agents (CWA) are, by nature, highly toxic. Additionally, sulfur‐containing CWA (*ie* sulfur mustards and V‐series nerve agents) are very stable with long and detrimental persistency in the environment, and it is imperative to avoid any transmission in the soil when transferring the toxic liquid for neutralisation.^[1]^ The key sulfur atom plays a major role in these negative effects: the ‘mustard gas’ (yperite, HD) is in equilibrium with the very electrophilic –and therefore highly toxic– episulfonium form formed through the neighbouring‐assistance of this very sulfur atom. In V‐series nerve agents, the pivotal −S−(CH_2_)_2_−N(Alk)_2_ fragment offers a moderate leaving group ability and therefore a good stability, together with a good acetycholine hydrolysis transition state mimick.^[2]^ Actually, in both cases, this “noxious sulfur atom” can also be viewed as the Achilles’ heel of these CWAs by taking advantage of its ability to undergo oxidation (Figure 1).

For sulfur mustards, interrupting the anchimeric assistance by oxidation into the harmless sulfoxide **1** is a well‐established method, provided that no over‐oxidation into the sulfone occurs.[^3^, ^4^, ^5^, ^6^, ^7^] The latter indeed undergoes fast dehydrochlorination that affords highly toxic vinylsulfone **2** (Figure 1a).[^8^, ^9^, ^10^, ^11^] In V‐series agents, the aminoethanethiol moiety is a poor leaving group (Figure 1b), responsible for its persistent properties and reluctance to hydrolysis. In 1990, Yang demonstrated that the oxidation‐assisted neutralisation of VX was a very effective method to afford innocuous ethyl methylphosphonate **4 a**, as long as the oxygen transfer occurs only on the sulfur atom and not on the more reactive nitrogen atom.[^12^, ^13^] The *N*‐oxidation product typicaly undergoes a Claisen rearrangement with release of an hydroxylamine derivative and of toxic vinyl thiophosphonate EA‐2192 **3 a** (Figure 1b).

For VX, the acidic oxidant Oxone® (2KHSO_5_⋅KHSO_4_⋅K_2_SO_4_) was shown to be an efficient oxidant by protonating the amino group while transferring oxygen atoms onto the sulfur.[^12^, ^13^, ^14^] However, in the development of sustainable detoxification methods, it is highly desirable to use oxidizing agents that generate as little effluents as possible,[^15^, ^16^] and aqueous hydrogen peroxide (H_2_O_2_) is much appealing: it is inexpensive, easy to handle and water is the only effluent.^[17]^ However, aq. H_2_O_2_ is a poor oxidant by itself and the combination with a catalyst to generate a stronger oxidizing agent is required in the case of CWA remediation. For such a purpose, biocatalysis is very attractive and some scarce examples of hydrogen peroxide combined with chloroperoxidase^[18]^ and microperoxidase AcMP11^[19]^ have been described. Recently, it has been reported that *Candida antarctica* lipase B (CALB, sold on solid support under the tradename of Novozym®) could generate the powerful peracetic acid AcOOH from H_2_O_2_ and AcOEt.^[20]^ Interestingly, immobilized (bio)catalysts exhibit the advantage to be suitable for classical batch‐ as well as packed‐bed flow reactors.[^21^, ^22^, ^23^, ^24^, ^25^, ^26^, ^27^, ^28^] Continuous‐flow reactors consist of reacting chemicals through narrow channels (often of submillimetric internal dimensioning).[^29^, ^30^] The intrinsic features of this technology make them very appealing for chemical synthesis, especially for the safe generation/handling of hazardous compounds such as oxidants,[^31^, ^32^, ^33^, ^34^] with successful application in sulfur oxidation.[^35^, ^36^] Therefore, flow devices are appealing safe systems for the controlled oxidative neutralisation of CWA, since the selectivity of the process is a key aspect to afford harmless compounds. This has been successfully performed with commercial oxidizing reagents[^14^, ^37^] or by generating more reactive/unstable oxidants upstream in the flow system.[^38^, ^39^, ^40^] Here the ‘on demand’ biocatalyzed generation of the hazardous peracetic acid would be of high interest.^[41]^

Along these lines, we report the remediation of sulfur mustard‐ and VX‐simulants, CEES and PhX,^[42]^ mediated by supported lipase CALB under batch and flow conditions.

## Results and Discussion

### Oxidative Neutralisation of CEES under Batch Conditions

First experiments on the oxidative neutralisation of the mustard simulant CEES into sulfoxide CEESO were performed under batch conditions with on the shelf reagents CALB, ethyl acetate and hydrogen peroxide (Table 1).

**Table 1:** CALB‐assisted oxidation of CEES with hydrogen peroxide/AcOEt under batch conditions.^[a]^

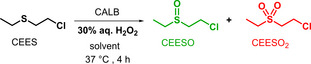					
Entry	Solvent	V (mL)	[CEES] mmol/L	Aq. H_2_O_2_ (equiv.)	Conv (%)^[b]^
1	AcOEt	5	200	2	90
2	AcOEt	10	100	2	63
3	AcOEt/EtOH (8 : 2)	10	100	2	72
4	AcOEt/EtOH (8 : 2)	10	100	3	73
5	AcOEt/EtOH (8 : 2)	5	200	2	91
6^[c]^	AcOEt/EtOH (8 : 2)	5	200	2	97
7	AcOEt/*t*‐amyl alcohol (1 : 1)	5	200	2	84
8	AcOEt/MeCN (8 : 2)	25	40	2	59

[a] Experiments were performed with 1 mmol of CEES and 25 mg CALB over 4 h. [b] Full selectivity toward desired sulfoxide CEESO (monitored by GC). [c] Performed with 37.5 mg of CALB over 6 h.

As stated above, AcOEt as solvent is mandatory since it is the source of AcOOH and each experiment was thus performed with 1 mmol of CEES in the presence of AcOEt as a solvent over 4 h and heated at 37 °C to keep the enzyme stable (25 mg) and at its optimal operating temperature. To maximize successful transposition under flow conditions, we targeted only liquid reagents and thus focused on 30 % aq. H_2_O_2_ as hydrogen peroxide source. With 2 equiv. of the primary oxidant in 5 mL of AcOEt, 90 % conversion into the desired sulfoxide (CEESO) was afforded after 4 h, with no undesired overoxidation into toxic CEESO_2_, nor any other side products (entry 1). Two‐fold increase of the amount of AcOEt (10 mL), as peracetic acid precursor, proved detrimental (63 % conversion), probably due to dilution effect (entry 2). One of the limits of such medium is that reaction occurs at the interface of three phases: the organic phase containing the sulfur compound and the ester reagent, the aqueous phase carrying hydrogen peroxide (AcOOH being partitioned between the two phases) and the solid biocatalyst. In order to limit mass transfer issues that affect reaction effectiveness in batch and in flow, our attention focused on the full solubilisation of aq. H_2_O_2_ in the reaction medium. Ethanol was then added as co‐solvent (8 : 2 AcOEt/EtOH ratio, 10 mL) allowing to reach a two‐phase system with a single liquid phase and a solid phase. Thus, the conversion reached 72 % (entry 3), whereas increasing the amount of H_2_O_2_ (3 equiv.) did not show further improvement (73 %; entry 4). By applying a two‐fold increase of the concentration in the same solvent mixture (5 mL), 91 % of CEESO were obtained (entry 5). Increasing the reaction time to 6 h afforded almost full conversion (97 %, entry 6). Since EtOH could negatively impact the lipase activity due to the reversibility of the transformation of AcOEt (vide infra Figure 3), sterically hindered *t*‐amyl alcohol was assessed to replace this co‐solvent. However, this led to a decreased conversion (84 %, entry 7), probably due to sluggish competitive lipase‐catalyzed esterification of released AcOH with the cumbersome *t*‐amyl alcohol. With an 8 : 2 AcOEt/MeCN mixture (also solubilizing all liquid reagents), the conversion dropped to 59 % (entry 8). This latter result is not surprising since acetonitrile has already been shown to partially poison CALB.^[43]^ As expected, it is worth to note that in the absence of CALB, no reaction occurred.

### Oxidative Neutralisation of CEES under Flow Conditions

**Figure 2:**
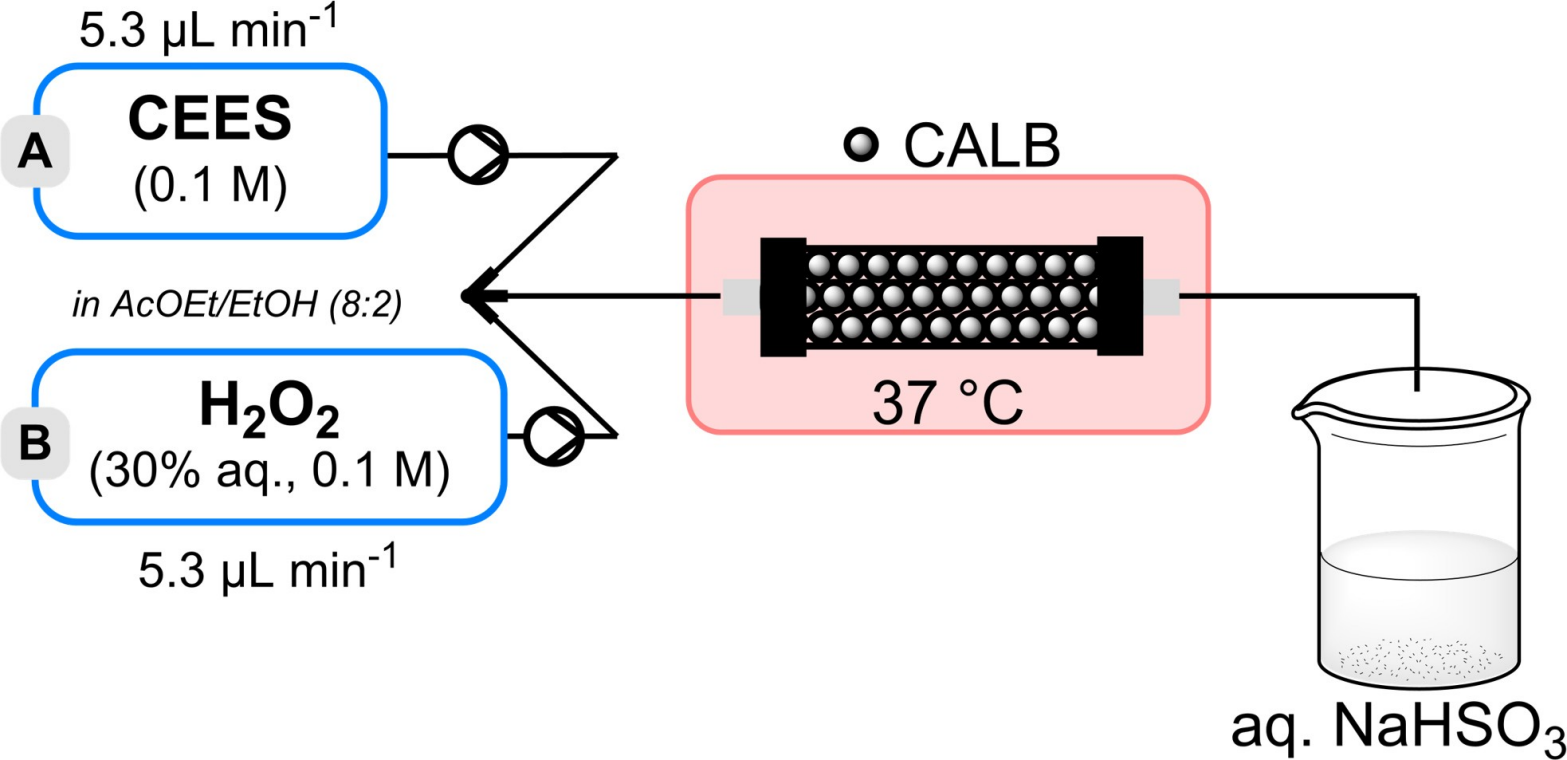
Simplified flow chart for the continuous oxidative detoxification of CEES using CALB/hydrogen peroxide/AcOEt.

Based on the preliminary results obtained under batch conditions, a flow set‐up with 2 inlets was implemented as follows: inlet A containing CEES and inlet B containing 30 % aq. H_2_O_2_, both reagents dissolved in AcOEt/EtOH (8 : 2). These inlets fed a packed‐bed reactor (PBR) filled with CALB and the outlet was connected to a quench solution of sodium bisulfite (Figure 2).

Interestingly, flow systems allow a single set‐up to assess various conditions, especially by modifying the relative or the total flow rate to tune, respectively, the relative stoichiometry or the reaction time (residence time within the reactor, *t* ^R^). Thus, the residence time was arbitrary set to 1 h with a PBR containing 100 mg of CALB (Table 2). With 2 equiv. of aq. H_2_O_2_ full conversion of CEES (C=0.1 M) was afforded but, surprisingly, the selectivity into desired sulfoxide was only 42 % accompanied by 58 % of undesired sulfone. This could be due to a relative higher enzyme amount in the packed bed reactor (compared to the batch reactor), but also very low flow rate (*Q*_T_=10.6 μL/min) may induce stagnation zones with longer contact time between enzyme and reactants. To increase the selectivity toward CEESO, the residence time was lowered to 30 min. This led to a slight increase of the selectivity (59 %) but a lower conversion (39 %, Entry 2). As expected, increasing the amount of H_2_O_2_ from 2–4 equivalents mainly overoxidized the substrate to CEESO_2_ (Entry 3). Raising the amount of CALB in the PBR to 200 mg did not have any positive influence to reach the target values (entries 4–6). In contrast, it appeared that there is a balance to be found between the concentration of sulfide, the amount of H_2_O_2_ and that of CALB. With [CEES]=0.01 M and 200 mg of CALB, it was possible to maintain full conversion into the desired CEESO, the conversion showed to be associated to the relative amount of H_2_O_2_, from 45 % (1.0 equiv.)–99 % (1.8 equiv.) as detailed in entries 7–10. The same efficiency was observed with 150 mg of CALB (entries 11–13). Delightfully, with [CEES]=0.1 M and only 1 equiv. of H_2_O_2_ full conversion into CEESO was afforded.

**Table 2:** CALB‐assisted oxidation of CEES with hydrogen peroxide/AcOEt under flow conditions.^[a]^

Entry	CALB (mg)	[CEES] (mmol/L)	Aq. H_2_O_2_ (equiv.)	Conv. (%)	CEESO (%)^[b]^
1	100	100	2	>99	42
2^[c]^	100	100	2	39	59
3	100	100	4	>99	6
4	200	100	1	49	99
5	200	100	2	>99	43
6	200	100	4	>99	31
7	200	10	1	45	>99
8	200	10	1.2	57	>99
9	200	10	1.5	66	>99
10	200	10	1.8	99	>99
11	150	10	2	98	>99
12	150	10	2.2	>99	96
13	150	10	2.5	>99	96
14	**150**	**100**	**1**	**>99**	**>99**

[a] Experiments were performed with *t* ^R^=1 h. [b] Only CEESO and CEESO_2_ were formed. [c] *t* ^R^=0.5 h.

Finally, the potential deactivation of the enzyme under these conditions was assessed by monitoring the outlet concentration in CEES and CEESO with time‐on‐stream. It showed that the CALB bed could be used for >100 h in a row and the activity began to decrease from 110 h (see Supporting Information for details).

### Oxidative Neutralisation of PhX under Batch Conditions

Due to the presence of the oxidizable nitrogen that could potentially yield toxic thiophosphonate **3 b**, it is important to find adequate conditions that circumvent this competitive oxidation path, such as acidic conditions promoting the protonation of the nitrogen atom. Therefore, it was expected that the (per)acetic acid species formed during the biocatatalysed process could protonate and deactivate the nitrogen atom and then favour the desired sulfur‐oxidation path. The results are reported in Table 3 (0.091 mmol scale of PhX; 4 h reaction time).[^13^, ^14^]

**Table 3:** CALB‐assisted oxidation of CEES with hydrogen peroxide/AcOEt under batch conditions.^[a]^

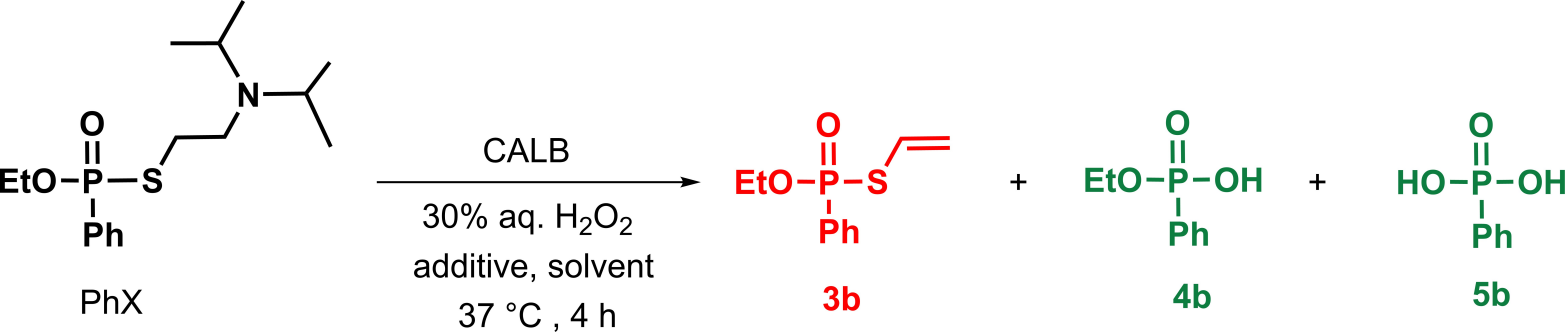						
Entry	Solvent	CALB (mg)	Aq. H_2_O_2_ (equiv.)	Additive (equiv.)	Conv. (%)^[b]^	Product distribution **3b**/**4b**/**5b** ^[b]^
1	AcOEt/EtOH (8 : 2)	6	6.7	/	>99	85 : 9 : 6
2	AcOEt/EtOH (8 : 2)	18	6	/	>99	99 : 0 : 0
3	AcOEt/EtOH/AcOH (7 : 2 : 1)	18	14	/	58	26 : 14 : 60
4	AcOEt/EtOH/AcOH (7 : 2 : 1)	18	14	HFIP (2)	59	42 : 9 : 49
5	**AcOEt/AcOH (9 : 1)**	**18**	**14**	**HFIP (2)**	**>99**	**0 : 100 : 0**
6	AcOEt	18	14	HFIP (2)	>99	39 : 61 : 0
7	AcOEt/AcOH (9 : 1)	0	14	HFIP (2)	23	0 : 99 : 0
8	AcOEt/EtOH (8 : 2)	18	0	/	0	/

[a] Reactions were performed with 0.091 mmol of PhX in 5 mL of solvent, 4 h reaction time. [b] Measured by ^31^P NMR.

Even if the mechanism of the oxidative decontamination through sulfur oxidation remains debated, it is admitted that >3 equiv. of oxidizing agents are required since the released sulfur‐containing side group ends up as sulfonic acid (Figure 1). Therefore, our first experiment was performed with an excess of aq. H_2_O_2_ (>6 equiv./PhX) and 6 mg of CALB in classical medium AcOEt/EtOH (8 : 2). Monitoring by ^31^P NMR showed complete disappearance of the starting PhX (*δ*=45.4 ppm) and led to the formation of three main products (Table 3, entry 1). A ^31^P NMR signal at 41.8 ppm, typical of a thiophosphonate structure, and likely corresponding to undesired product **3 b** as the major product (85 %) as confirmed by analyses. The two other main signals were associated with innocuous products **4 b** (9 %; *δ*=20.7 ppm) and **5 b** (4 %; *δ*=18.9 ppm). Unfortunately, increasing the amount of CALB (18 mg) further amplified the formation of **3 b** (entry 2). These two experiments suggest that the reaction medium was not acidic enough to protonate the nitrogen atom and prevent undesired *N*‐oxidation.

**Figure 3:**
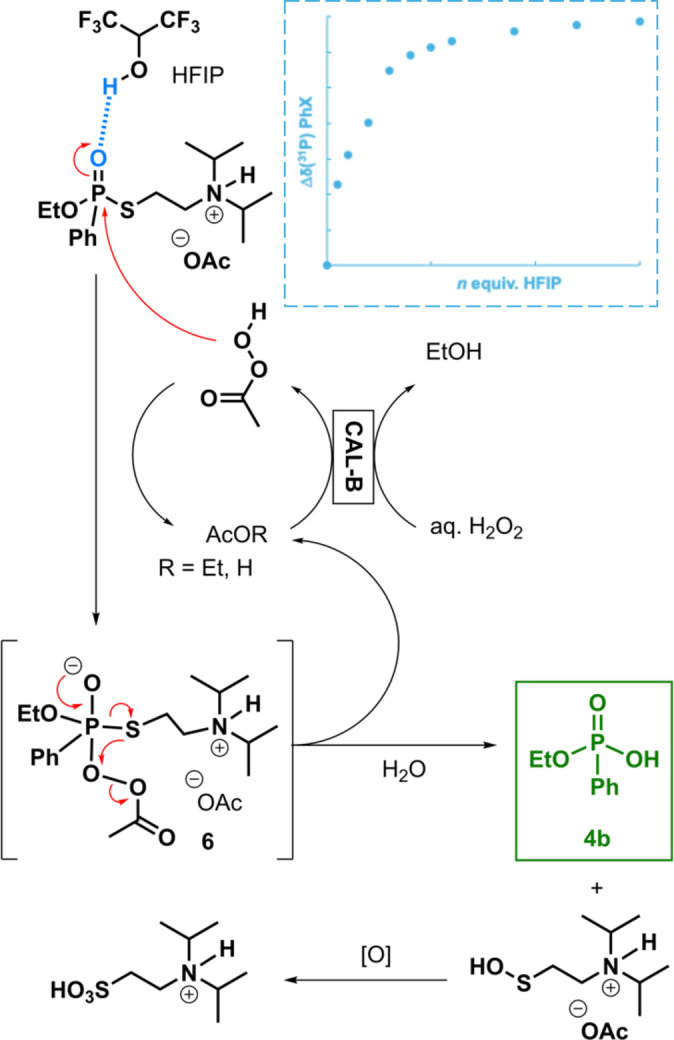
Proposed mechanism for the CALB‐mediated remediation of VX‐simulant PhX with aq. H_2_O_2_/AcOET assisted by AcOH and HFIP.

As a mitigation measure, AcOH was directly added to the solvent composition (entry 3). Under these conditions, phosphonothioate **3 b** was not the main product anymore, but still produced in significant amount (26 %), but the conversion dropped to 58 %, even with a larger excess of H_2_O_2_ (14 equiv.). Consequently, HFIP ((CF_3_)_2_CHOH, 2 equiv.), a very strong H‐bond donor, was considered as a potential additive. Indeed, HFIP's ability to form Lewis acid‐base complexes could lead to a synergic dual action both by deactivation of PhX's nitrogen and activation of PhX's P=O).[^44^, ^45^] Unfortunately, no positive effect was observed, probably due to the competitive H‐bond interactions with EtOH which is also a good H‐bond acceptor (entry 4).^1^ Removing EtOH from the solvent mixture indeed gave full conversion and selectivity toward ethyl phenylphosphonic acid **4 b** (entry 5). Using pure AcOEt as solvent (by removing also AcOH) proved detrimental with full disappearance of PhX but with significant formation of **3 b** (39 %) along with **4 b** (61 %), as shown in entry 6. Therefore, it is clear that both AcOH and HFIP are necessary for as successful oxidative neutralisation: through their respective actions on preventing *N‐*oxidation and the activation of the P=O bond. To validate the essential role of each component of the neutralisation solution, two control experiments were carried out. Without CALB, only 23 % conversion were attained, affording the uncatalyzed hydrolysis process formation of **4 b**. When aq. H_2_O_2_ and HFIP were removed, no reaction occurred. To support the role of HFIP, NMR experiments were performed by adding an increasing amount of the fluorinated alcohol on a solution of PhX. Monitoring the P phosphonothioate ^31^P NMR shift, providing a significant association constant *K*=*ca*. 3.6 M^−1^ (Figure 3).^2^

On the basis of these results, a tentative mechanism is proposed in Figure 3. In the reaction medium containing acetic acid, the diisopropylaminoethyl side chain of PhX is protonated, while HFIP binds the oxygen atom of the P=O group, increasing thus the electropositive character of the phosphorus atom, and thus favouring the next addition/elimination steps. The peracetic acid formed with CALB/AcOEt/H_2_O_2_ can thus act as nucleophile and form intermediate 6. Elimination of the aminoethylthiol chain leaving group led to the corresponding sulfenic acid and to phosphonic acid **4 b**. The fate of the aminoethylsulfenic acid leaving group in the presence of an excess oxidizer eventually led to the corresponding sulfonic acid.

### Oxidative Neutralisation of PhX under Flow Conditions

**Figure 4:**
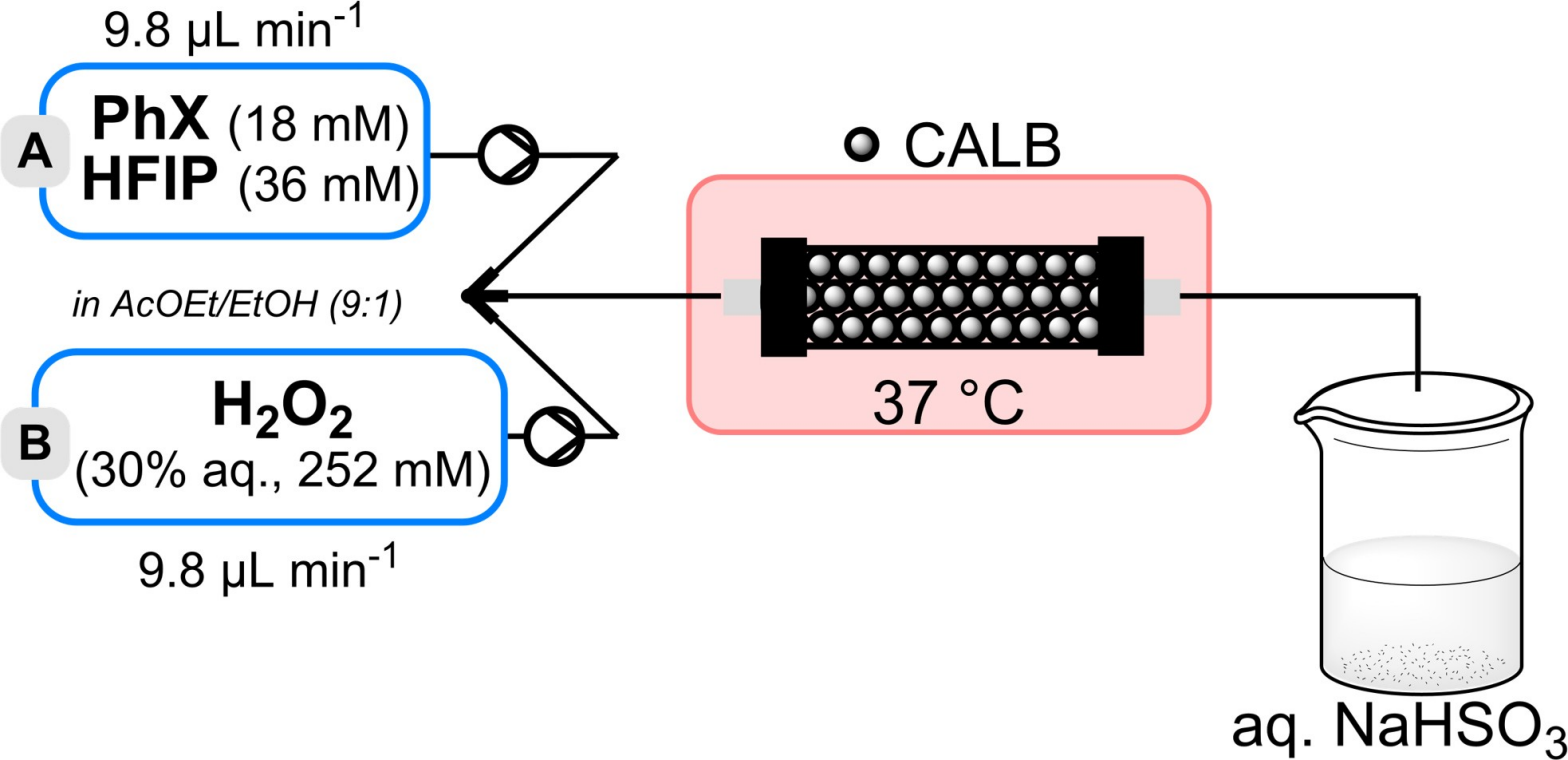
Simplified flow chart for the continuous oxidative detoxification of PhX using CALB/hydrogen peroxide/AcOEt and HFIP.

Based on these batch results, and on those previously obtained in the flow oxidation reaction transposition with CEES, and taking into account that peracetic acid acts as a nucleophile and not strictly as an oxidant, thus allowing working with a high lipase amount, the detoxification of PhX, was attempted in a flow reactor (Figure 4).

Thus, a 2‐inlet flow system was implemented on the same basis as for CEES (vide supra) but with [PhX]=18 mmol/L and the PBR containing 400 mg of CALB. With *t* ^R^=1 h, reaction proceeded as expected, affording a >99 % conversion of PhX and >99 % selectivity toward the desired phosphonic acid **4 b**. It should be noted that we did not observe any adsorption of the substrate onto the acrylic resin of PMMA in batch or flow conditions. However, this could happen (with real CWA for example) the limited contact time between liquid and solid reagents in flow should make this the preferred approach.

## Conclusions

We have shown that the supported lipase CALB, commercially available, efficiently assists the oxidative detoxification of sulfur‐containing CWA in the presence of green and affordable aq. H_2_O_2_ and AcOEt. For the sulfur mustard simulant CEES, the presence of EtOH as co‐solvent favours both efficiency and selectivity toward harmless CEESO. Regarding PhX, a simulant of VX, its inherent structural features required different conditions to drive both efficiency and selectivity of the neutralisation process. Thus, replacing EtOH by AcOH as co‐solvent allowed to deactivate the competitive path starting with nitrogen‐oxidation, while a stoichiometric addition of H‐bond donor HFIP in the medium facilitated the selective decomposition into innocuous ethyl phosphonic without any toxic byproduct. An inexpensive, widely available supported lipase was conveniently packed in an off‐the‐shelf column without any additional treatment. It affords a cheap, effective and widely transposable tool to perform safe and on‐demand remediation of each sulfur‐containing CWA simulants through *in situ* generation of peracetic acid.

In conjunction with common and low environmental footprint reagents (AcOEt, EtOH, AcOH and aq. H_2_O_2_), this work represents a significant breakthrough in regards of the current state of the art, where expensive enzymes are generally used for the detoxification of such simulants.

## Experimental Section

### General Information

Samples of sulfur compounds were analyzed by gas chromatography coupled to flame ionisation detection (Thermo Scientific™ TRACE™ 1310 GC‐FID). Molecular structures identity was confirmed by GC‐MS. Samples of phosphorus compounds were identified using ^31^P NMR high field analyses on a 400 MHz Bruker Spectrospin spectrometer. 2‐Chloroethyl ethyl sulfide (CEES), immobilized Candida Antarctica lipase B (CALB sold as Novozym®435), ethanol, ethyl acetate, 30 % aqueous hydrogen peroxide, water, and sodium thiosulfate were purchased from commercial sources and used without additional purification. 2‐chloroethyl ethyl sulfone (CEESO_2_), 2‐chloroethyl ethyl sulfoxide (CEESO) and S‐{2‐[Di(propan‐2‐yl)amino]ethyl} O‐ethyl phenylphosphonothioate (PhX) were prepared according to protocols from the literature.[^37^, ^42^]

All fluidic tubing, connections, and adapters were ordered from Upchurch‐IDEX Health and Science. Syringe pumps were purchased from Harvard apparatus (Pump 11 Elite and PHD/ULTRA) loaded with HENKE‐JECT® Luer Lock plastic syringes. Feed and collection lines consisted of PFA tubing (1/16′′ o.d., 1.0 mm i.d.). Flow oxidation was carried out using an OmnifitTM column filled with Novozym®435 beads as packed bed reactor (PBR).

### Safety Statement

CAUTION: mustard gas (HD) and S‐{2‐[di(propan‐2‐yl)amino]ethyl} O‐ethyl methylphosphonothioate (VX) are both subjected to heavy military restriction on possession and study that any user should be aware of, and only OPCW (Organisation for the Prohibition of Chemical Weapons) accredited laboratories are allowed to possess limited amounts for detoxification studies. 2‐Chloroethyl ethyl sulfide (CEES) is the most common simulant of HD and is widely accepted at purchase and study. But 2‐chloroethyl ethyl sulfide (CEES) is still a highly toxic and severe vesicant and must be handled with caution. All contaminated glassware should be neutralized with bleach prior to disposal (be warned that the quench solution containing sodium thiosulfate is chemically not compatible with bleach). PhX is the simulant with the closest chemical reactivity to that of VX and still remains a toxic nerve agent and must be handled with caution. All contaminated glassware should be neutralized by soaking in a bleach solution for 24 hours prior to disposal.

### Typical Procedure for the Oxidative Neutralisation of CEES in Batch

The typical run was carried out in a 25 mL round‐bottomed flask. CALB (20 % w/w, 25 mg) was added to a solution of CEES (1 equiv., 1 mmol, 116 μL) in an AcOEt/EtOH solution (8 : 2, 5 mL). The mixture was then stirred at 37  °C for 5 min. 30 % Aq. H_2_O_2_ (2 equiv., 2 mmol, 204 μL) was finally added to trigger the reaction and the mixture was stirred at 37 °C for 6 h. 50 μL aliquots were regularly taken from the crude and placed in a vial containing 1950 μL of isopropanol (IPA). The obtained sample was then dried over MgSO_4_ and filtrated to remove any trace of water and salt. After, 50 μL of each sample was diluted with 950 μL of isopropanol and was added 20 μL of internal standard solution (internal standard solution: 50 μL n‐decane in 950 μL IPA) for GC‐FID analysis.

### Typical Procedure for the Oxidative Neutralisation of CEES in Flow

**Packed‐bed preparation**: the Omnifit^TM^ column was filled with 150 mg CALB and weighted (m_1_). 5 mL of AcOEt was then injected in the dry enzyme‐filled column at 250 μL/min using a syringe pump to remove any air from the CALB PBR. The column was weighed again (m_2_). The internal volume of the reactor was then determined as follows:

$$V_{internal}=\;\frac{m_{2}-m_{1}}{\rho_{AcOEt}}$$

CEES flow oxidation protocol: the CALB PBR was washed with 5 mL of AcOEt/EtOH (8 : 2, v/v) at 250 μL/min using a syringe pump. Then, the syringe pump was loaded with two 5 mL‐syringes filled containing feeding solutions. The first one with a 0.1 mol/L solution of CEES in AcOEt/EtOH (8 : 2). The second one with a 0.1 mol/L solution of aq. 30 % H_2_O_2_ in AcOEt/EtOH (8 : 2). The PBR was then immersed into a water bath at 37 °C for 15 min to reach the temperature equilibrium then both solutions were injected into the reactor (Q_1_=Q_2_=5.3 μL/min i. e. Q_T_=10.6 μL/min; t^R^=1 h). After 4 h, the collection of the crude was started in a vial containing 150 μL of a solution of sodium bisulfite to quench any trace of peroxide. The obtained crude was then dried over MgSO4 and filtrated to remove any trace of water and salt and 50 μL was sampled and diluted it with 950 μL of isopropanol. 20 μL of internal standard solution was added (internal standard solution: 50 μL n‐decane in 950 μL isopropanol) for GC analysis.

### Typical Procedure for the Oxidative Neutralisation of PhX in Batch

The typical run was carried out in a 25 mL round‐bottomed flask. CALB (20 %w/w, 6 mg) was added to a solution of PhX (1 equiv., 9.1⋅10^−5^ mol, 28 μL) in a AcOEt/EtOH solution (8 : 2, 5 mL). The mixture was then stirred at 37 °C for 5 min. 30 % aq. H_2_O_2_ (14 equiv., 1.3 mmol, 140 μL) was finally added to start the reaction and stirred at 37 °C for 6 h. 333 μL aliquots were regularly taken from the crude and put in an NMR tube containing 300 μL of CDCl_3_ and an external standard solution of 85 % H_3_PO_4_ in D_2_O for ^31^P NMR analysis.

### Typical Procedure for the Oxidative Neutralisation of PhX in Flow

**Packed‐bed preparation**: the Omnifit^TM^ column was filled with 400 mg CALB and weighed (m_1_). 5 mL of AcOEt was then injected in the dry enzyme‐filled column at 250 μL/min using a syringe pump to remove any air of the inside form the CALB PBR. The column was weighed again (m_2_). The internal volume of the reactor was then determined as follows:

$$V_{internal}=\;\frac{m_{2}-m_{1}}{\rho_{AcOEt}}$$

PhX flow oxidation protocol: the CALB packed bed was washed with an AcOEt/AcOH solution (9 : 1, 5 mL) at 250 μL/min using a syringe pump. Then, the syringe pump was loaded with two 5 mL syringes filled with feeding solutions. The first one with a solution of PhX (1 equiv., 18 mM) and HFIP (2 equiv., 36 mM) in AcOEt/AcOH (9 : 1). The second one with a solution of 30 % aq. H_2_O_2_ (14 equiv., 252 mM) in AcOEt/AcOH (9 : 1). The PBR was immersed in a water bath at 37 °C for 15 min to reach the temperature equilibrium then both solutions were injected into the reactor (Q_1_=Q_2_=9.8 μL/min i. e. Q_T_=19.6 μL/min; *t* ^R^=1 h). After 4 h, the collection of the crude was started in a vial containing 150 μL of a solution of sodium bisulfite in 150 μL of H_2_O to quench any trace of peroxide. 333 μL aliquots were regularly taken from the crude and put in an NMR tube containing 300 μL of CDCl_3_ and an external standard solution of 85 % H_3_PO_4_ in D_2_O for ^31^P NMR analysis.

## Supporting Information Summary

This manuscript's experimental data (experimental details, characterization, ^1^H, ^31^P and ^13^C NMR copies, and GC data are available in the ESI. The authors have cited additional references within the Supporting Information.[^46^, ^47^]

## Conflict of Interests

The authors declare no conflict of interest.

1

## Supporting information

As a service to our authors and readers, this journal provides supporting information supplied by the authors. Such materials are peer reviewed and may be re‐organized for online delivery, but are not copy‐edited or typeset. Technical support issues arising from supporting information (other than missing files) should be addressed to the authors.

Supporting Information

## Data Availability

The data that support the findings of this study are available in the supplementary material of this article.

## Footnotes

n1 Hydrogen‐bonding acceptance properties (*β*) of related compounds taken from reference:^[48]^ MeO_3_P=O (0.65)>EtOH (0.62)>AcOEt (0.52)>AcOH (0.38)>H_2_O (0.37)>HFIP (0.16).

n2 Estimated with BindFit software: http://app.supramolecular.org/bindfit/
