# Supplementary material for: Oxidative Neutralisation of Sulfur‐Based Chemical Warfare Agents Mediated by a Lipase: From Batch to Flow Reactor
Source: Chemistry. 2025 Mar 12;31(19):e202403701. doi: 10.1002/chem.202403701 (PMC11962350; doi:10.1002/chem.202403701)
Supplement: Supplementary file 1 — Supporting Information [file CHEM-31-e202403701-s001.pdf]

Chemistry–A European Journal

Supporting Information

Oxidative Neutralisation of Sulfur-Based Chemical Warfare Agents Mediated by a Lipase: From Batch to Flow Reactor

Maxime Boddaert, Valmir Baptista da Silva, Sergui Mansour, Daniela Vuluga, Pierre-Yves Renard, Jean-Christophe M. Monbaliu,* and Julien Legros*

Supporting information for

Oxidative neutralisation of sulfur-based chemical warfare agents mediated by a lipase: from batch to flow reactor

Maxime Boddaert,[a,b] Valmir Baptista da Silva,[a] Sergui Mansour,[a] Daniela Vuluga,[c] Pierre-Yves Renard,[a] Jean-Christophe M. Monbaliu*[b,d] and Julien Legros*[a]

a. Univ Rouen Normandie, INSA Rouen Normandie, CNRS, Normandie Univ, COBRA UMR 6014, F-76000 Rouen, France
Julien.legros@univ-rouen.fr | www.lab-cobra.fr

b. Center for Integrated Technology and Organic Synthesis, MolSys Research Unit, University of Liège, B-4000 Liège (Sart Tilman), Belgium
jc.monbaliu@uliege.be | www.citos.uliege.be

c. INSA Rouen Normandie, Univ Rouen Normandie, CNRS, PBS 76000 Rouen, France
www.pbs.cnrs.fr

d. WEL Research Institute, Avenue Pasteur 6, B-1300 Wavre (Belgium)

1. SAFETY STATEMENT 3

2. GENERAL INFORMATION 3

2.1. CHEMICALS AND FLOW MATERIALS 3
2.2. FLOW MATERIALS 4
MICROFLUIDIC SETUP AND PARTS 4
Pumps 4
Connectors, ferrules, unions 4
Packed-bed cartridge 4
TABLE OF PARTS AND VENDORS 4
SCHEMATIC DEPICTION OF GENERAL THE FLOW SETUP 4
2.3. ANALYTICAL METHODS 5

3. BATCH SYNTHESIS, PROTOCOLS AND RESULTS 7

3.1. BATCH OXIDATION OF CEES AND RESULTS 7
3.2. BATCH SYNTHESIS OF REFERENCES 7
3.2.1. Synthesis of 2-chloroethyl ethyl sulfoxide standard 7
3.2.2. Synthesis of 2-chloroethyl ethyl sulfone standard 8
3.2.3. Synthesis of PhX 10
*3.2.4. Synthesis of phenyl phosphinic acid monoethyl ester standard **4b** 10*
3.3. BATCH OXIDATION OF PHX AND RESULTS 11

4. FLOW SYNTHESIS, PROTOCOLS AND RESULTS 12

4.1. FLOW OXIDATION OF CEES AND RESULTS 12
4.2. TIME-ON-STREAM NEUTRALIZATION OF CEES INTO CEESO WITH CALB PACKED BED REACTOR 14
4.3. FLOW OXIDATION OF PHX AND RESULTS 15

5. CHARACTERIZATION OF STARTING MATERIALS AND COMMERCIAL REFERENCES 16

5.1. 2-CHLOROETHYL ETHYL SULFIDE (CEES) 16
5.2. PHX 17
5.3. PHENYLPHOSPHINIC ACID (**5B**) 19
5.4. MONOETHYL PHENYLPHOSPHONATE (**4B**) 19
5.5. O-ETHYL S-VINYL PHENYLPHOSPHONOTHIOATE (**3B**) 20

6. ASSOCIATION OF HFIP AND PHX MEASURED BY ^{31}P NMR 21

7. REFERENCES 22

1. Safety statement

CAUTION: 2-Chloroethyl ethyl sulfide (CEES) and PhX are highly toxic and must be handled with caution (PPE under a well-ventilated fume hood). All contaminated glassware should be neutralized by soaking in a bleach solution for 24 hours prior to disposal. (be warned that the quench solution containing sodium thiosulfate is chemically not compatible with bleach).

2. General information

2.1. Chemicals and flow materials

Chemicals and solvents purchased from commercial sources were used without additional purification.

Solvents	Purity (%)	CAS Number	Supplier
Absolute Ethanol	≥99.8	64-17-5	Fisher Scientific
Acetic acid	≥99	64-19-7	Sigma Aldrich
Ethyl acetate	≥99	141-78-6	Fisher Scientific
Propan-2-ol	≥99.5	67-63-0	Fisher Scientific
DI water			
Chemicals	Purity (%)	CAS Number	Supplier
Sodium thiosulfate	99%	7772-98-7	Sigma Aldrich
UHP	97%	124-43-6	Aldrich
Hydrogen peroxide solution 30% wt	30%	7722-44-1	Sigma Aldrich
Novozym®435		9001-62-1	Merck
2-chloroethyl ethyl sulfide	97%	693-07-2	Sigma Aldrich
Hexafluoroisopropan-2-ol	99%	920-66-1	Sigma Aldrich
Phenylphosphinic acid	98%	1779-48-2	Thermo scientific
Diethyl phenylphosphonate	96%	1754-49-0	TCI

2.2. Flow materials

Microfluidic setup and parts

All microfluidic setups were assembled with commercially available parts.

Pumps

Harvard apparatus (Pump 11 Elite and PHD/ULTRA) loaded with 5 mL HENKE-JECT® Luer Lock plastic syringes or 100 mL TRAJAN® Luer Lock SGE glass syringes.

Connectors, ferrules, unions

PFA coils were assembled both with coned PEEK fittings or Super Flangeless PEEK nuts and ETFE ferrules from IDEX. Mixers consisted of PEEK Tee-mixers (0.02" through hole) or PEEK cross-junctions (0.02" trough hole).

Packed-bed cartridge

The packed-bed reactor was composed of an Omnifit™ column equipped with two 30 µm PTFE frits filled with Novozym®435 immerged in a thermostated bath.

Table of parts and vendors

Table S 1: Parts list for fluidic elements and connectors.

Items	Details	Vendor	Reference
Connectors	SuperFlangeless™ Male Nut 1/16in PEEK	INACOM Instruments/IDEX	P-255
	SuperFlangeless™ Ferrule Assembly 1/16''	INACOM Instruments/IDEX	P-259
	FingerTight I PEEK	INACOM Instruments/IDEX	F-120
Mixers	Static Mixing Tees with F-300 Fingertight Fittings for 1/16" OD tubing	INACOM Instruments/IDEX	U-466
Unions	Union body 1/4-28 - 1/16'' PEEK	INACOM Instruments/IDEX	P702-01
Tubing	Tubing PFA High Purity 1/16" OD, 0.020" ID (50 ft)	INACOM Instruments/IDEX	1622L

Schematic depiction of general the flow setup

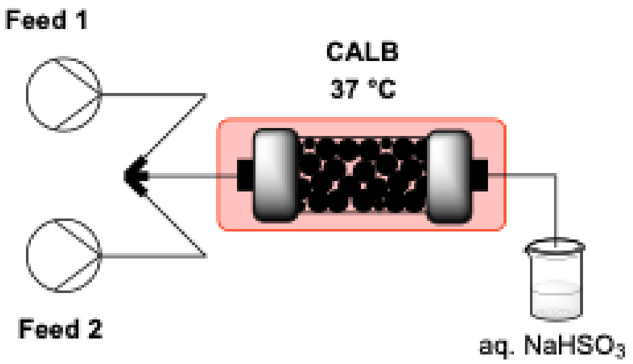

Scheme S 1: Schematic depiction of the microfluidic setup for the decontamination of CEES and PhX.

2.3. Analytical methods

Samples of sulfur compounds were analyzed by gas chromatography coupled to flame ionisation detection (Thermo Scientific™ TRACE™ 1310 GC-FID). Molecular structures identity was confirmed by GC-MS. Samples of phosphorus compounds were identified using ^{31}P NMR high field analyses on a 300 or 400 MHz Bruker Spectrospin spectrometer.
GC analyses were conducted with a Thermo Scientific™ TRACE™ 1310 gas chromatograph equipped with a flame-ionization (FID) detector and Zebron ZB-5 phenomenex capillary column (30 m × 0.25 mm i.d., 0.25 µm). GC-MS analyses were conducted with a Thermo Scientific™ TRACE™ 1310 gas chromatograph equipped with a flame-ionization (FID) detector, Zebron ZB-5 phenomenex capillary column (30 m × 0.25 mm i.d., 0.25 µm).
High field ^{1}H, ^{31}P and ^{13}C NMR studies were performed on a 300 MHz or 400 MHz Bruker Spectrospin spectrometer. Chemical shifts (δ) are given with regard to TMS using solvent as internal reference, *J* coupling constants are given in Hertz.

Elution gradient for GC-FID analysis.

Hold Time (min)	Rate (°C/min)	T (°C)
2	0	50
25	50	250
2	0	250

3.

Total time: 12 min

Elution gradient for GC-MS.

Hold Time (min)	Rate (°C/min)	T (°C)
2	0	50
14	25	250
2	0	250

4.

Total time: 18 min

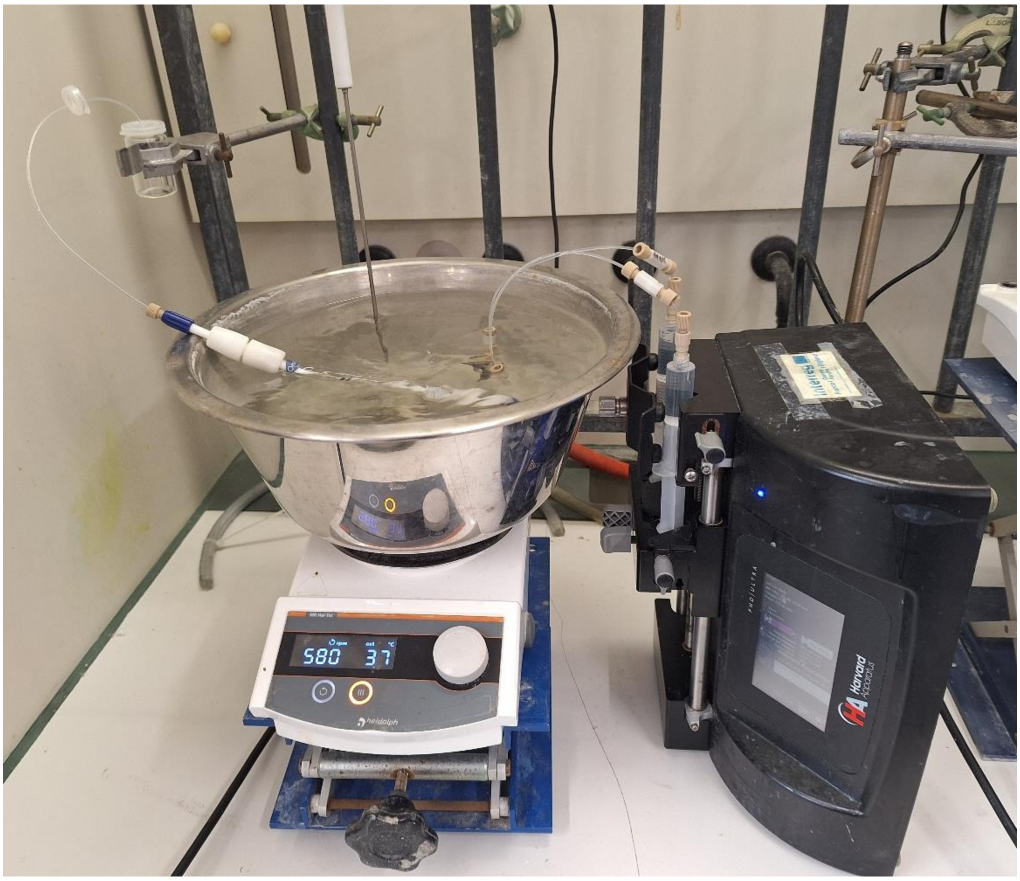

Figure S 1: Flow set up for the oxidation of CEES and PhX.

3. Batch synthesis, protocols and results

3.1. Batch oxidation of CEES and results

The typical run was carried out in a 25 mL round flask. CALB (20% w/w, 25 mg) was added to a solution of CEES (1 equiv, 1 mmol, 116 µL) in 5 mL AcOEt/EtOH 8: 2. The mixture was then stirred at 37 °C for 5 min. H_2O_2 (2eq, 2 mmol, 204 µL) was finally added to start the reaction and stirred at 37 °C for 6 h. 50 µL was regularly sampled from the crude and placed in a vial containing 1950 µL of isopropanol. The obtained sample was then dried over $MgSO_4$ and filtrate to remove any trace of water and salt. Then, 50 µL of each sample was diluted with 950 µL of IPA and 20 µL of internal standard solution were added (internal standard solution: 50 µL n-decane in 950 µL IPA) for GC-FID analysis.

Table S 2: Oxidation of 1 mmol CEES (1 eq) with CALB lipase assisted with peroxide in batch condition

Entry	Solvent	CALB mass (mg)	Volume (mL)	[CEES] mmol/L	Peroxide	Peroxide equivalent	Reaction time (h)	Conversion (%)
**1**	AcOEt	25	5	200	UHP	1.1	2	62
**2**	AcOEt	25	5	200	UHP	2	2	38
**3**	AcOEt/EtOH (1 :1)	25	5	200	UHP	1.1	4	18
**4**	AcOEt/EtOH (1 :1)	25	5	200	UHP	2	4	28
**5**	AcOEt	25	5	200	H_2O_2	2	4	90
**6**	AcOEt	25	15	67	UHP	2	4	78
**7**	AcOEt	25	10	100	H_2O_2	2	4	63
**8**	AcOEt/EtOH (8 :2)	25	10	100	H_2O_2	2	4	72
**9**	AcOEt/EtOH (8 :2)	25	10	100	H_2O_2	3	4	73
**10**	AcOEt/EtOH (8 :2)	25	5	200	H_2O_2	2	4	91
**11**	**AcOEt/EtOH (8 :2)**	**37.5**	**5**	**200**	**H_2O_2**	**2**	**6**	**97**
**12**	AcOEt/Amyl alcohol (1 :1)	25	5	200	H_2O_2	2	4	84
**13**	AcOEt/MeCN (8 :2)	25	25	40	H_2O_2	2	4	59

3.2. Batch synthesis of references

3.2.1. Synthesis of 2-chloroethyl ethyl sulfoxide standard

CEES (1 equiv., 4 mmol, 433 µL) was dissolved in 4 mL of AcOH in a 25 mL round flask. A solution of H_2O_2 30% aq (2 equiv., 8 mmol, 819 µL) was added and the mixture was stirred for 30 min at room temperature. After that the reaction was quenched with a solution of sodium bisulfite 40% v/v and the crude was extracted using dichloromethane and brine and evaporated under high vacuum. The crude was then analyzed by ^1H NMR and GC-FID.[37]

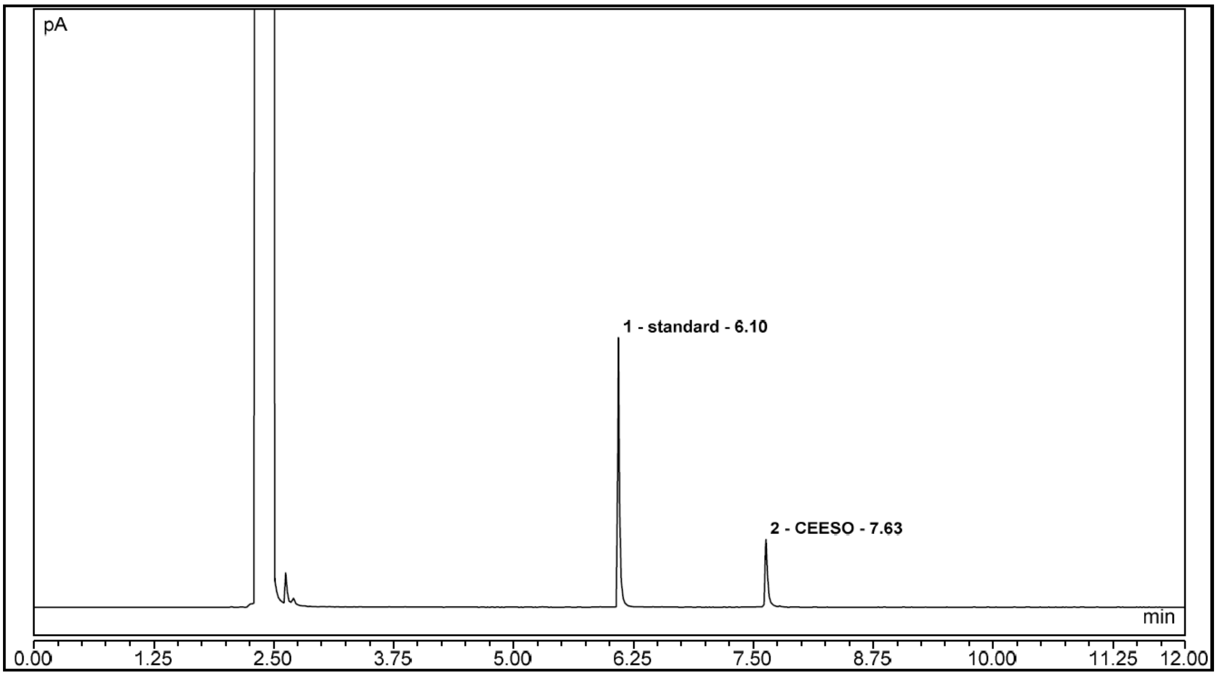

Figure S 2: GC-FID chromatogram of 2-chloroethyl ethyl sulfoxide.

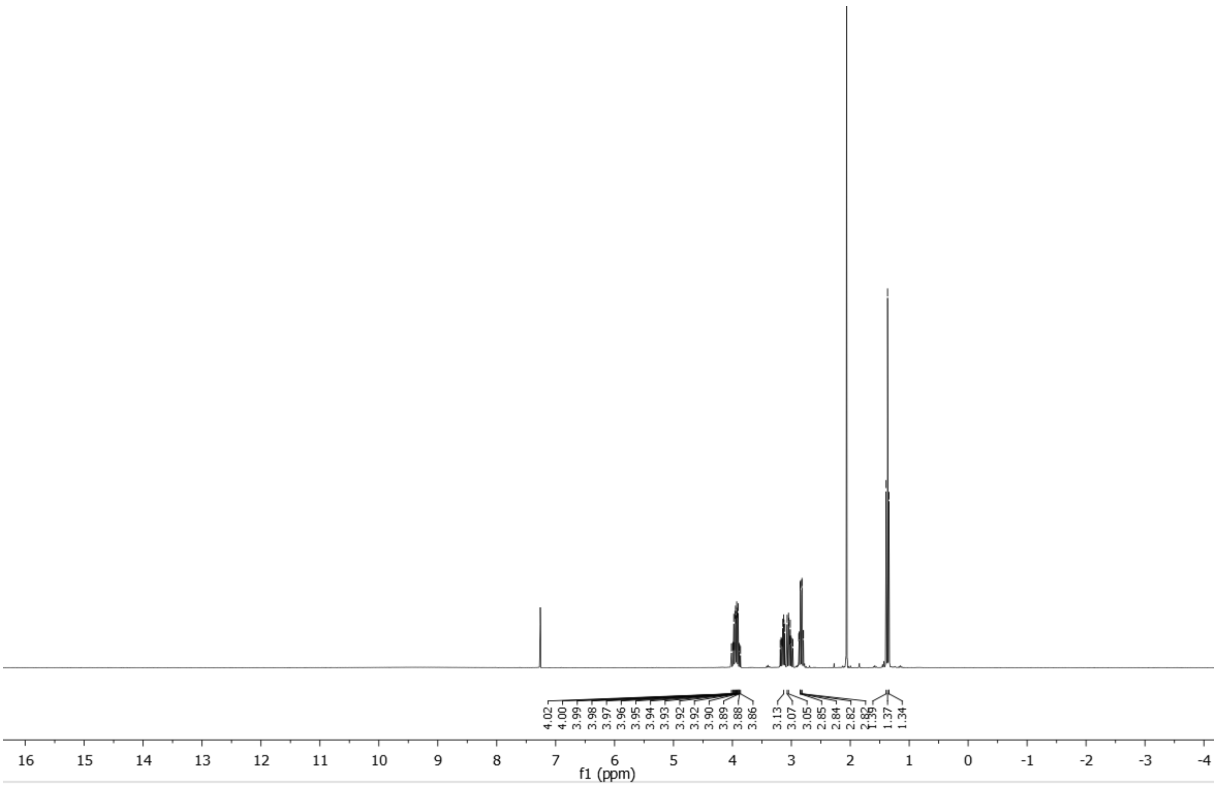

Figure S 3: ^1H NMR spectrum (400 MHz) of 2-chloroethyl ethyl sulfoxide in $CDCl_3$.

3.2.2. Synthesis of 2-chloroethyl ethyl sulfone standard

$KMnO_4$ was dissolved in 20 mL of H_2O/MeCN (1:3, v/v) in a 50 mL round flask, CEES (1 equiv., 0.5 mmol, 59 µL) was added. The mixture was then cool down to 0 °C and the 1.1 mL of concentrated H_2SO_4. After that the crude was stirred for 17 h at room temperature and quenched with a solution of sodium bisulfite 40 % and stirred until the color disappeared. The obtained mixture was then extracted with dichloromethane and evaporated under high vacuum. The crude was then analyzed by ^1H NMR and GC-FID.[37]

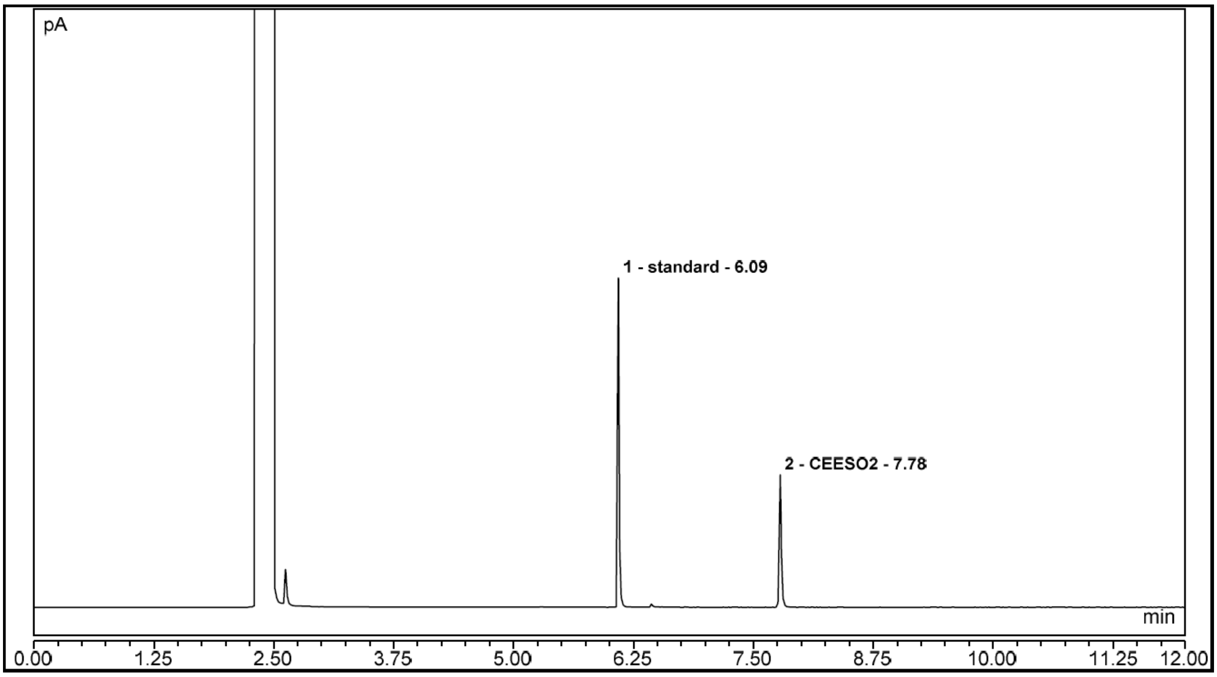

Figure S 4: GC-FID chromatogram of 2-chloroethyl ethyl sulfone.

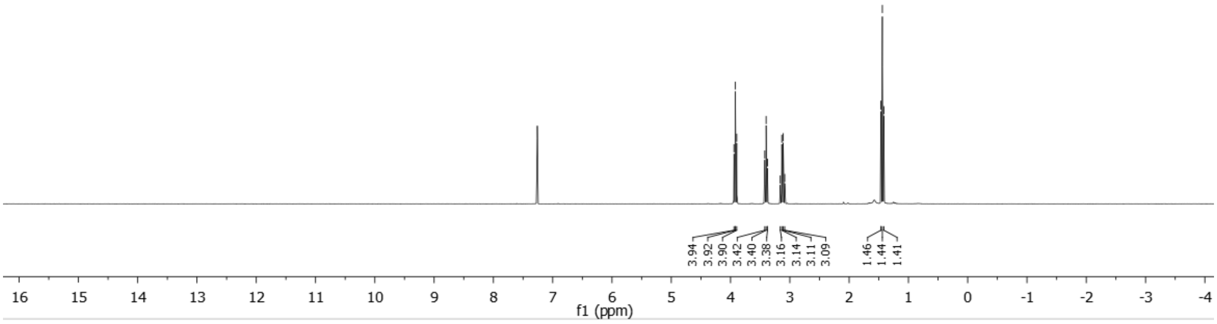

Figure S 5: ^{1}H NMR spectrum (400 MHz) of 2-chloroethyl ethyl sulfone in $CDCl_3$.

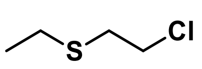 2-chloroethyl ethyl sulfide<br>C_4H_9ClS<br>MW = 124.63	**GC-FID:** 5.56 min. (**Figure S 7**)<br>**^{1}H NMR (300 MHz, CDCl$_3$) δ** 3.56 – 3.63 (m, 2H), 2.77–2.87 (m, 2H), 2.55 (q, J = 7.4 Hz, 1H), 1.23 (t, J = 7.4 Hz, 2H).<br>**^{13}C{^{1}H} NMR (101 MHz, CDCl$_3$)** δ 43.1, 33.8, 26.3, 14.9
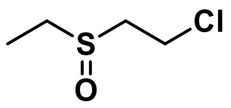 2-chloroethyl ethyl sulfoxide[37]<br>C_4H_9OClS<br>MW = 140.63	**GC-FID:** 7.63 min. (**Figure S 2**)<br>**^{1}H NMR (300 MHz, CDCl$_3$)** δ 4.04 – 3.85 (m, 2H), 2.89–3.03 (m, 2H), 2.83 (m, 2H), 1.37 (t, J = 7.5 Hz, 3H).<br>**^{13}C{^{1}H} NMR (75 MHz, CDCl$_3$)** δ 54.2, 46.1, 37.0, 6.8.
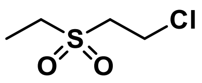 2-chloroethyl ethyl sulfone[37]<br>$C_4H_9O_2ClS$<br>MW = 156.62	**GC-FID:** 7.78 min. (**Figure S 4**)<br>**^{1}H NMR (300 MHz, CDCl$_3$)** δ 3.92 (t, J = 6.9 Hz, 2H), 3.40 (t, J = 6.9 Hz, 2H), 3.12 (q, J = 7.5 Hz, 2H), 1.44 (t, J = 7.5 Hz, 3H).<br>**^{13}C{^{1}H} NMR** (75 MHz, CDCl$_3$) δ 54.3, 49.1, 36.0, 6.7.

3.2.3. Synthesis of PhX

PhX was synthetized according to the literature.[42] The product was then analyzed by ^{31}P and ^{1}H NMR.

3.2.4. Synthesis of phenyl phosphinic acid monoethyl ester standard 4b

Phenyl phosphinic acid monoethyl ester was synthetized according to the literature.[46] The product was then analyzed by ^{31}P NMR.

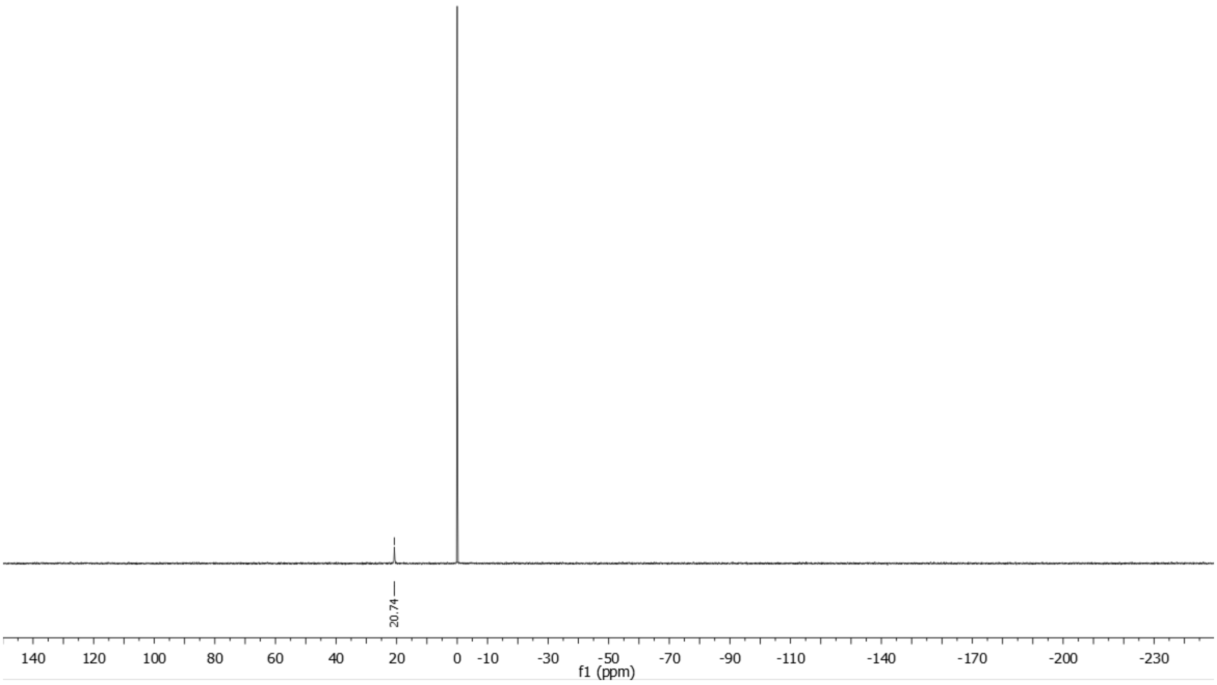

Figure S 6: ^{31}P NMR spectrum (121 MHz) of monoethyl phenylphosphonate acid **4b** in CDCl$_3$.

3.3. Batch oxidation of PhX and results

The typical run was carried out in a 25 mL round flask. CALB (20% w/w, 6 mg) was added to a solution of PhX (1 equiv., 9.1e-5 mol, 28 µL) in 5 mL AcOEt/EtOH 8: 2. The mixture was then stirred at 37 °C for 5 min. H_2O_2 (14 equiv., 1.3 mmol, 140 µL) was finally added to start the reaction and stir at 37 °C for 6 h. 333 µL was regularly sampled from the crude and placed in a NMR tube containing 300 µL of $CDCl_3$ and an external standard solution of phosphoric acid 85 % in deuterated water for ^{31}P NMR analysis.

Table S 3: Batch oxidation of 0.091 mmol PhX (1 equiv.) with CALB lipase assisted with peroxide in 5 mL of solvent, selectivity was measured by ^{31}P NMR peak integration.

Entry	Solvent	CALB mass (mg)	Peroxide equivalent	Additive	Conversion (%)	Selectivity
**1**	AcOEt/EtOH (8 :2)	6	6.7	/	>99	**1** 85%<br>**2** 9%<br>**3** 6%
**2**	AcOEt/EtOH (8 :2)	6	14	/	>99	**1** 81%<br>**2** 7%<br>**3** 12%
**3**	AcOEt/EtOH (8 :2)	18	6	/	>99	**1** >99%
**4**	AcOEt/EtOH (8 :2)	18	0	/	0	/
**5**	AcOEt/EtOH (8 :2)	18	4	AcOH (0.17 mM)	0	/
**6**	AcOEt/EtOH/AcOH (7 :2 :1)	18	14	/	58	**1** 26%<br>**2** 14%<br>**3** 60%
**7**	AcOEt/EtOH/AcOH (7 :2 :1)	18	14	HFIP (2 eq)	59	**1** 42%<br>**2** 9%<br>**3** 49%
**8**	**AcOEt/AcOH (9 :1)**	**18**	**14**	**HFIP (2 eq)**	**>99**	**2 >99%**
**9**	AcOEt/AcOH (9 :1)	18	4	HFIP (2 eq)	>99	**1** >99%
**10**	AcOEt	18	14	HFIP (2 eq)	>99	**1** 39%<br>**2** 61%
**11**	AcOEt/AcOH (9 :1)	0	14	HFIP (2 eq)	23	**2** >99%

Table S 4: Characterization of compounds for the oxidation PhX

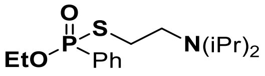<br>**PhX**	$C_{16}H_{28}NO_2PS$[47]<br>MW = 329.44	**$^{31}P\{^1H\}$ NMR (121 MHz, CDCl$_3$)** δ 46.06.<br>**^1H NMR (300 MHz, CDCl$_3$)** δ 7.92 - 7.81 (m, 2H), 7.56-7.40 (m, 3H), 4.23 – 32.89 (m, 2H), 2.93 (sept, *J*=6.6, 2H), 2.75 – 2.59 (m, 2H), 2.57 – 2.45 (m, 2H), 1.37 (t, *J*=7.1, 3H), 0.91 (dd, *J*=6.6, 1.4, 12 H)<br>**$^{13}C\{^1H\}$ NMR (101 MHz, CDCl$_3$)** δ 133.7 (d, J = 148.8) 132.06 (d, J = 3.2), 130.89 (d, J = 10.8), 128.15 (d, J = 14.7), 61.67 (d, J = 6.8), 48.47 (s), 45.86 (d, J = 4.7), 30.98 (d, J = 2.1), 20.51 (t, J = 6.1), 16.08 (d, J = 6.7)
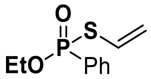<br>**3b**	$C_{10}H_{13}O_2PS$<br>MW = 228.04	**GC-FID/MS m/z :** 109.1; 125.1; 141.1; 142.1; 169.1; 170.1; 200.1; 228.2<br>**$^{31}P\{^1H\}$ NMR (121 MHz, CDCl$_3$)** δ 41.84.
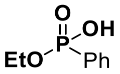<br>**4b**	$C_8H_{11}O_3P$[46]<br>MW = 186.15	**$^{31}P\{^1H\}$ NMR (121 MHz, CDCl$_3$)** δ 20.74.<br>**^1H (300 MHz, CDCl$_3$)** δ 71-7.87 (m, 2H), 7.46-7.55 (m, 1H), 7.34-7.46 (m, 2H), 4.04 (m, 2H), 1.26 (t, J = 7.09, 3H).<br>**$^{13}C\{^1H\}$ (75 MHz, CDCl$_3$)** δ 132.1 (d, J = 2.6), 131.38 (d, J = 10.2), 129.16 (d, J = 196), 128.3 (d, J = 15.2), 128.3 (d, J = 15.3), 61.9 (d, J = 6.6), 16.2 (d, J = 6.4.
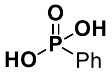<br>**5b**	$C_6H_7O_3P$*<br>MW = 158.09	**^{31}P NMR (121 MHz, CDCl$_3$)** δ 18.66. (**Figure S 13**)

4. Flow synthesis, protocols and results

4.1. Flow oxidation of CEES and results

Packed-bed preparation:

The column was filled with 150 mg CALB and weighted (m_1). 5 mL of AcOEt was then pumped in the dry enzyme filled column at 250 µL/min using a syringe pump to remove any air of the inside of the CALB bed. The column was weighted again (m_2). The internal volume of the reactor was then determined as following:

$$V_{internal} = \frac{m_2 - m_1}{\rho_{AcOEt}}$$

CEES flow oxidation protocol:

The CALB packed bed was washed with 5 mL of AcOEt/EtOH (8 :2 v/v) at 250 µL/min using a syringe pump. After that, the syringe pump was loaded with the two 5 mL syringes filled with feeds solutions. The first one with a 0.1 mol/L solution of CEES in AcOEt/EtOH (8 :2 v/v) in a 5 mL syringe. The second one with a 0.4 mol/L solution of H_2O_2 in AcOEt/EtOH (8 :2 v/v) in a 5 mL syringe. The packed bed was placed into a water bath at 37 °C for 15 min to reach the temperature equilibrium and the both feeds was pumped in the reactor. After 4 h, the collection of the crude was started in a vial containing 150

* Compared to authentic sample.

µL of a solution of sodium bisulfite to quench any trace of peroxide. The obtained crude was then dried over $MgSO_4$ and filtrate to remove any trace of water and salt and 50 µL was sampled and diluted it with 950 µL of IPA. 20 µL of internal standard solution was added (internal standard solution: 50 µL n-decane in 950 µL IPA) for GC analysis.

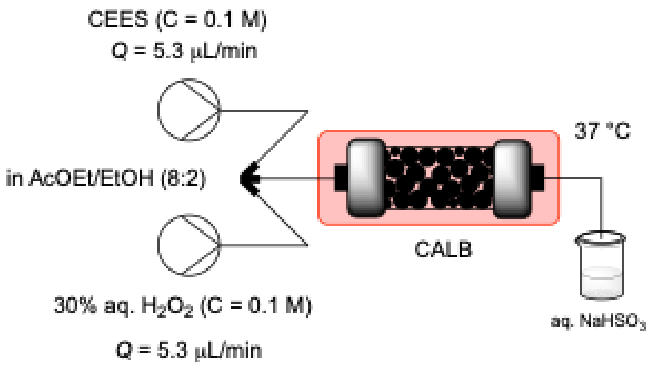

Table S 5: Flow oxidation of 1 mmol CEES (1 equiv.) with CALB assisted with peroxide

Entry	Residence time (min)	CALB mass (mg)	Bed volume (µL)	[CEES] mol/L	H_2O_2 equivalents	Conversion (%)	Selectivity in CEESO (%)
**1**	60	100	607	0.1	2	>99	42
**2**	30	100	575	0.1	2	39	59
**3**	60	100	662	0.1	4	>99	6
**4**	60	200	724	0.1	1	49	>99
**5**	60	200	768	0.1	2	>99	43
**6**	60	200	748	0.1	4	>99	31
**7**	60	200	743	0.01	1	45	>99
**8**	60	200	755	0.01	1.2	57	>99
**9**	60	200	783	0.01	1.5	66	>99
**10**	60	200	805	0.01	1.8	99	>99
**11**	60	150	673	0.01	2	98	>99
**12**	60	150	719	0.01	2.2	>99	96
**13**	60	150	678	0.01	2.5	>99	96
**14**	60	150	715	0.1	2	>99	40
**15**	**60**	**150**	**704**	**0.1**	**1**	**>99**	**>99**

4.2. Time-on-stream neutralization of CEES into CEESO with CALB packed bed reactor

Time (h)	% CEES	%CEESO
0	0	100
9	0	100
25	0	100
40	0	100
104	0	100
112	0	100
129	11.4	88.6

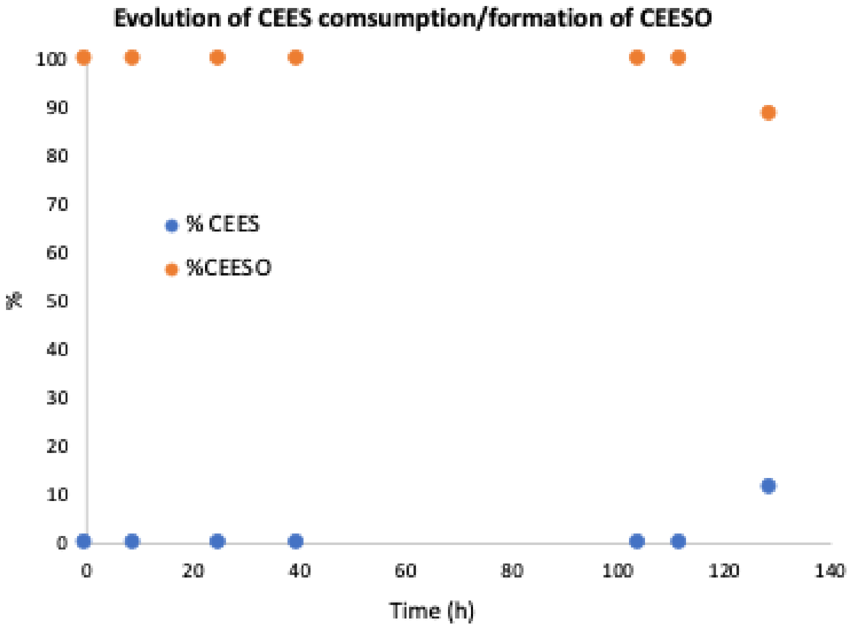

4.3. Flow oxidation of PhX and results

Packed-bed preparation:
The column was filled with 400 mg CALB and weighted (m_1). 5 mL of AcOEt was then pumped in the dry enzyme filled column at 250 µL/min using a syringe pump to remove any air of the inside of the CALB bed. The column was weighted again (m_2). The internal volume of the reactor was then determined as following:

$$V_{internal} = \frac{m_2 - m_1}{\rho_{AcOEt}}$$

PhX flow oxidation protocol:
the CALB packed bed was washed with an AcOEt/AcOH solution (9:1, 5 mL) at 250 µL/min using a syringe pump. Then, the syringe pump was loaded with two 5 mL syringes filled with feeding solutions. The first one with a solution of PhX (1 equiv., 18 mM) and HFIP (2 equiv., 36 mM) in AcOEt/AcOH (9:1). The second one with a solution of 30% aq. H_2O_2 (14 equiv., 252 mM) in AcOEt/AcOH (9:1). The PBR was immersed in a water bath at 37 °C for 15 min to reach the temperature equilibrium then both solutions were injected into the reactor (Q_1 = Q_2 = 9.8 µL/min i.e. Q_T = 19.6 µL/min; t^R = 1 h). After 4 h, the collection of the crude was started in a vial containing 150 µL of a solution of sodium bisulfite in 150 µL of H_2O to quench any trace of peroxide. 333 µL aliquots were regularly taken from the crude and put in an NMR tube containing 300 µL of $CDCl_3$ and an external standard solution of 85% H_3PO_4 in D_2O for ^{31}P NMR analysis.

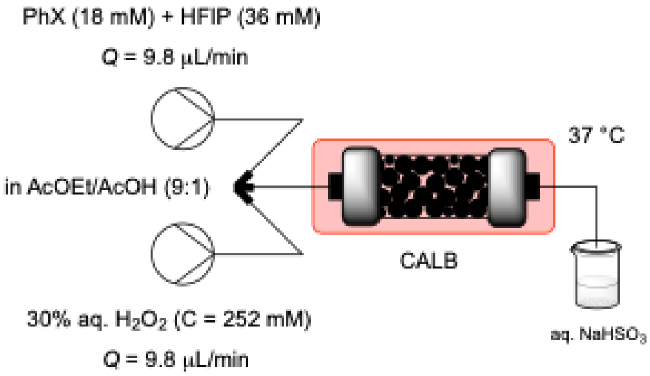

5. Characterization of starting materials and commercial references

5.1. 2-chloroethyl ethyl sulfide (CEES)

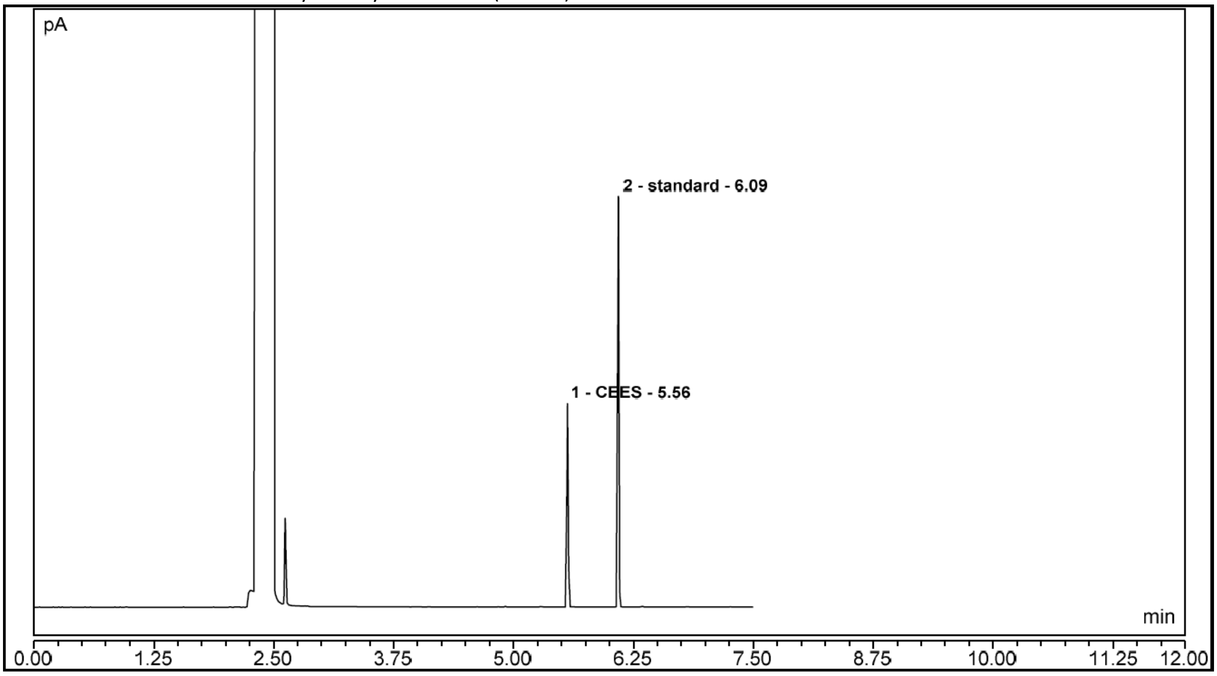

Figure S 7: GC-FID chromatogram of 2-chloroethyl ethyl sulfoxide.

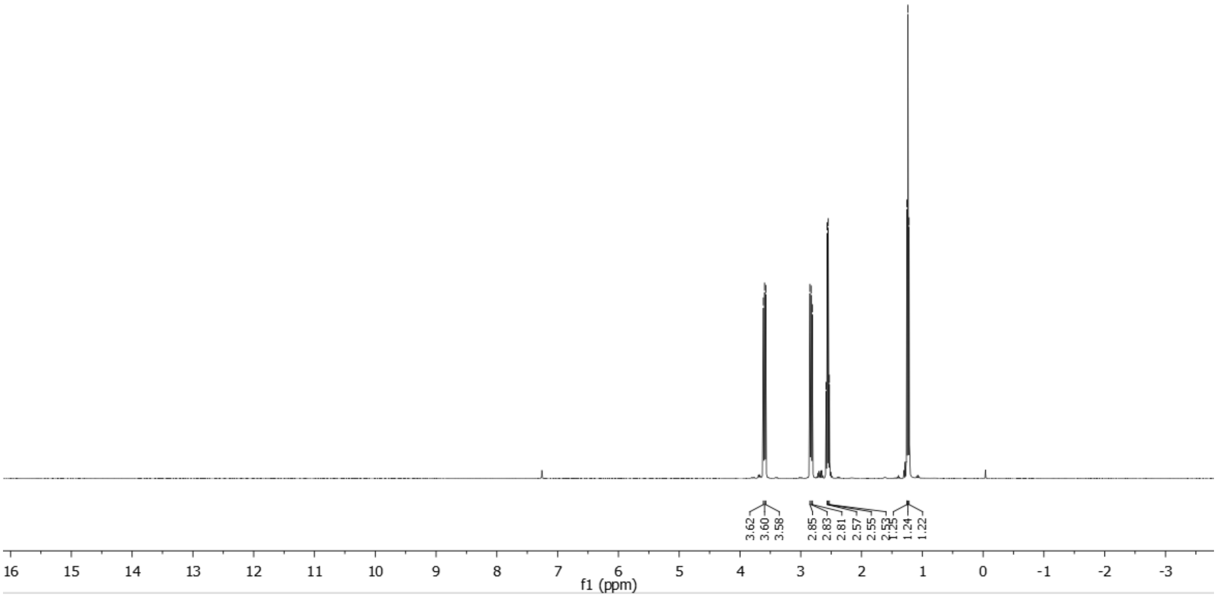

Figure S 8: ^{1}H NMR spectrum (300 MHz) of CEES in $CDCl_3$.

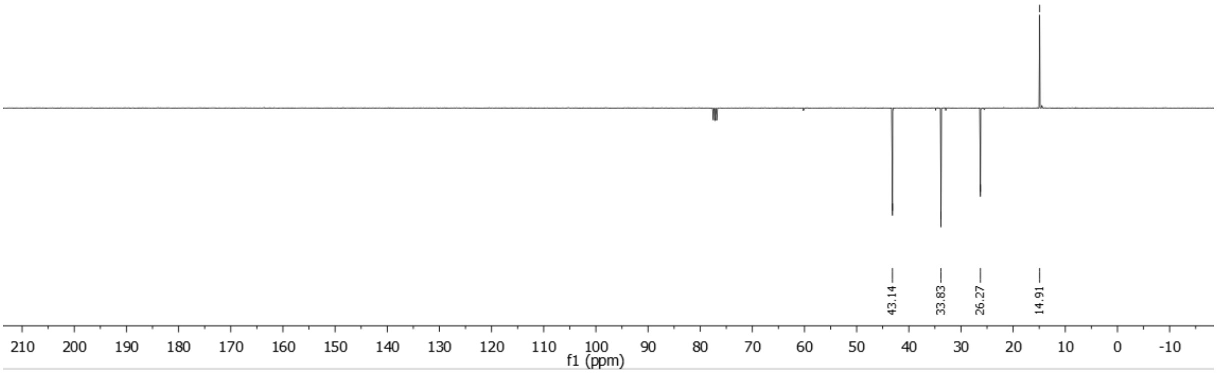

Figure S 9: $^{13}C\{^{1}H\}$ NMR (DEPT) spectrum (101 MHz) of CEES in $CDCl_3$.

5.2. PhX

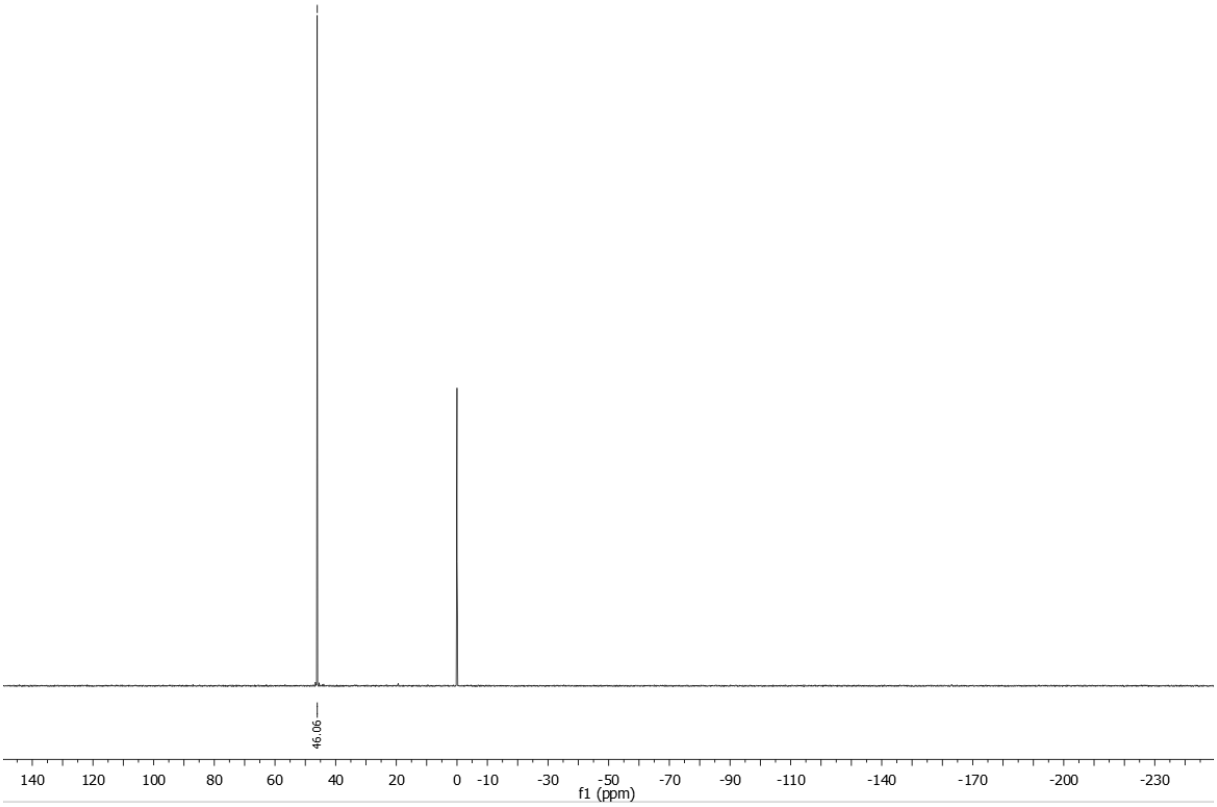

Figure S 10: $^{31}P\{^{1}H\}$ NMR spectrum (121 MHz) of PhX in $CDCl_3$.

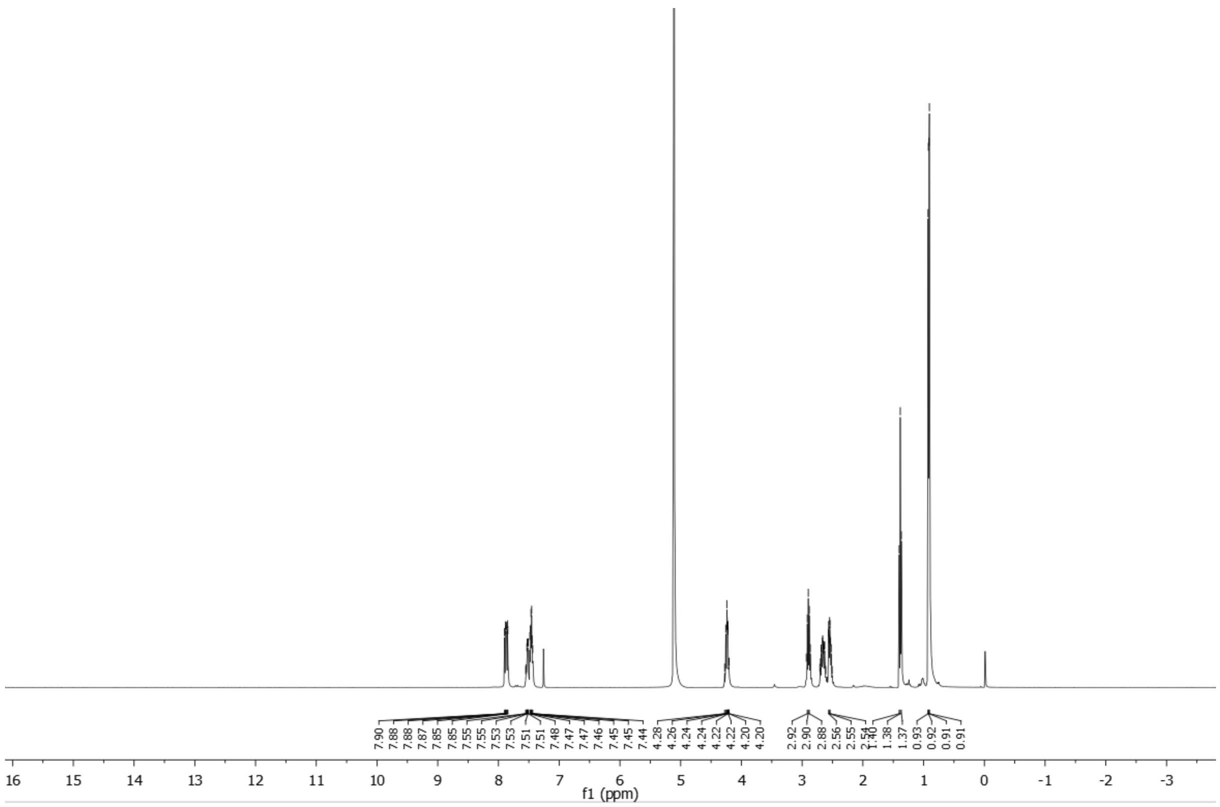

Figure S 11: 1H NMR spectrum (300 MHz) of PhX in $CDCl_3$.

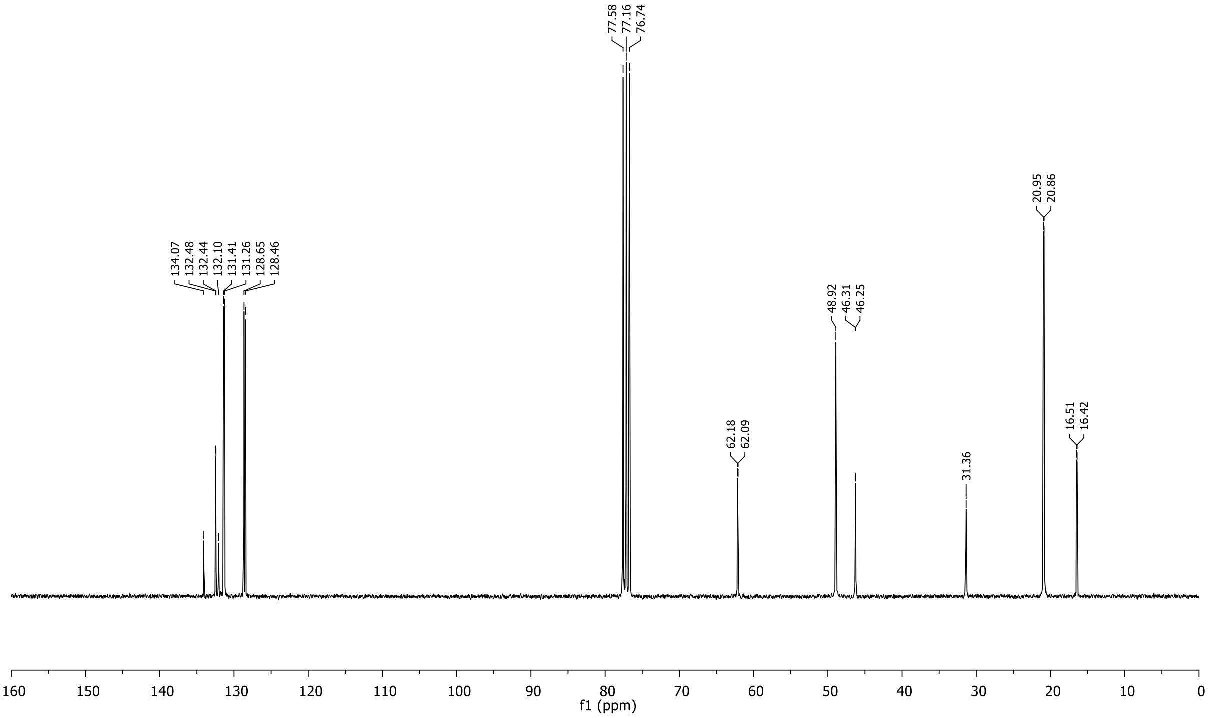

Figure S 12: $^{13}C\{^{1}H\}$ NMR spectrum (101 MHz) of PhX in $CDCl_3$.

5.3. Phenylphosphinic acid (**5b**)

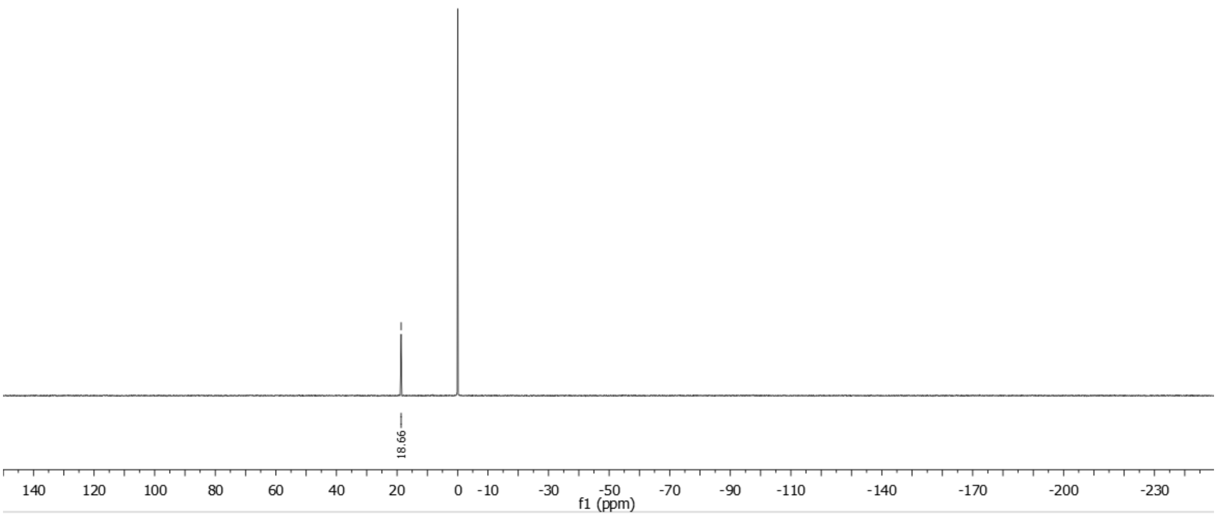

Figure S 13: $^{31}P\{^{1}H\}$ NMR spectrum (121 MHz) of phenylphosphinic acid **5b** in $CDCl_3$.

5.4. Monoethyl Phenylphosphonate (**4b**)

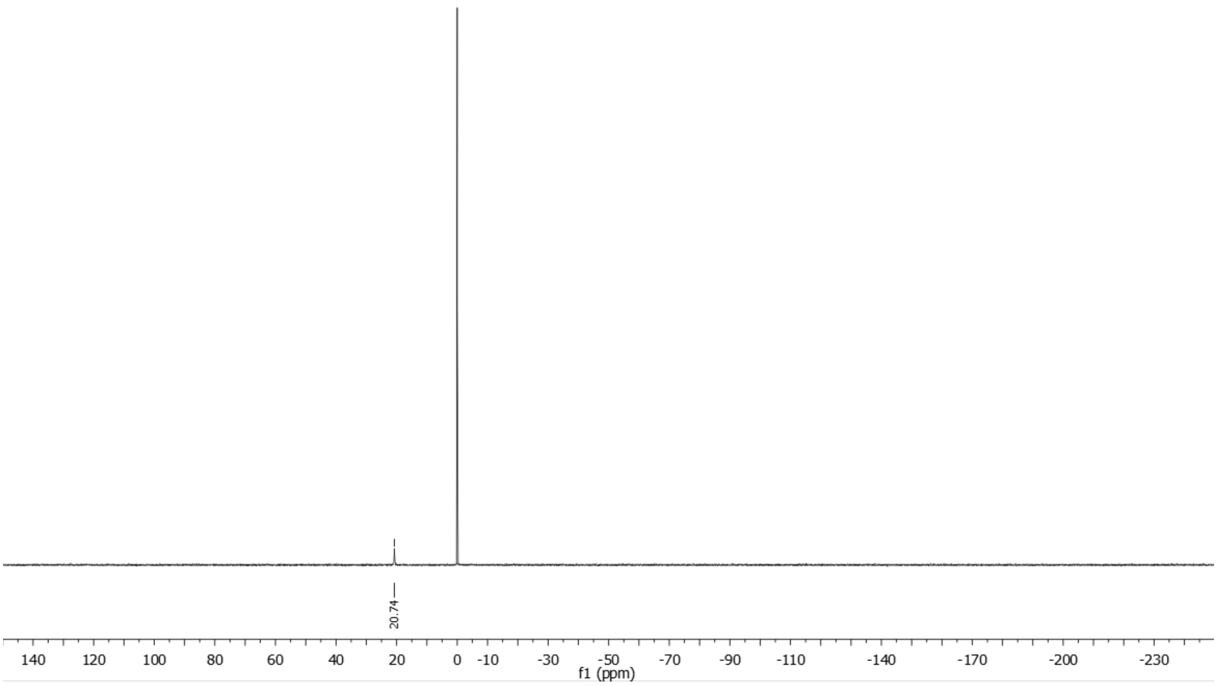

Figure S 14: $^{31}P\{^{1}H\}$ NMR spectrum (121 MHz) of monoethyl phenylphosphonate acid **4b** in $CDCl_3$.

5.5. O-Ethyl S-vinyl phenylphosphonothioate (3b)

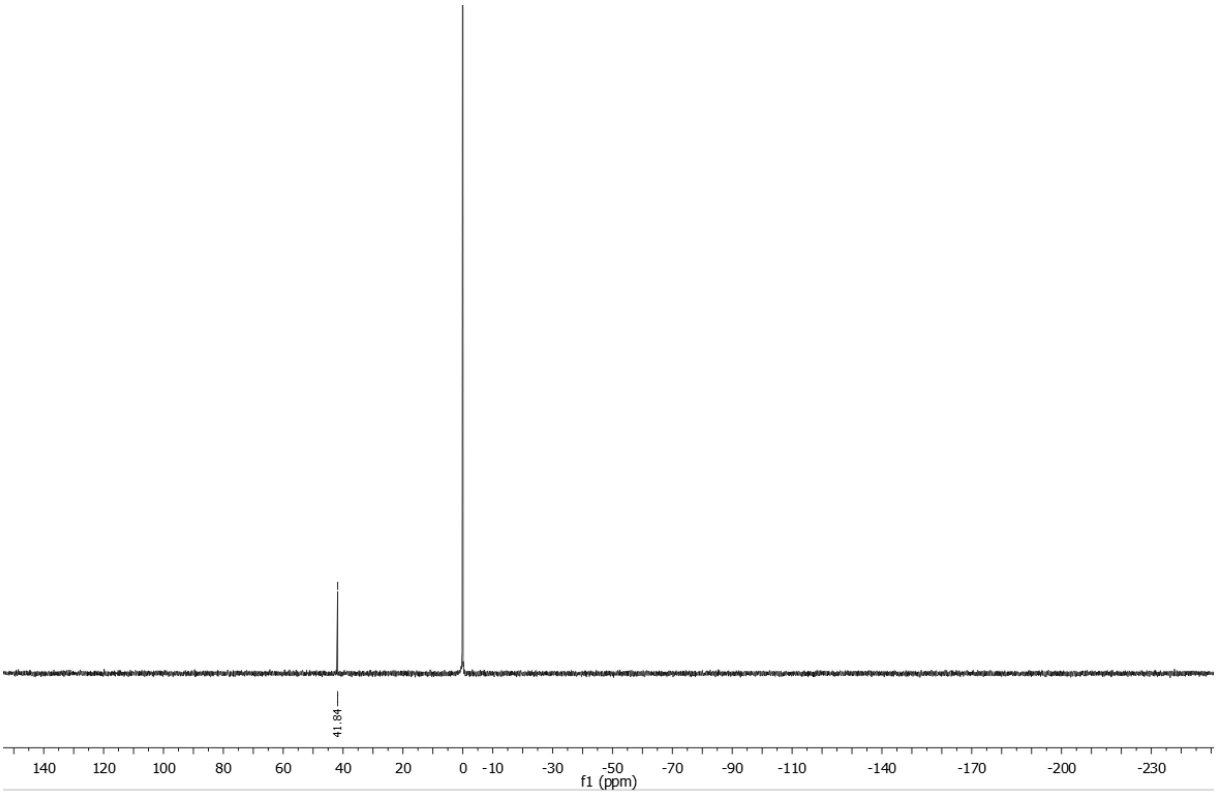

Figure S 15: $^{31}P\{^{1}H\}$ NMR spectrum (121 MHz) of O-ethyl S-vinyl phenylphosphonothioate **3b** in $CDCl_3$.

6. Association of HFIP and PhX measured by ^{31}P NMR

Nb eq HFIP	[HFIP]	δ(^{31}P) PhX	Δδ(^{31}P) PhX
0	0	46.02	0
1	0.12	48.3	2.28
2	0.24	49.13	3.11
4	0.48	50.04	4.02
6	0.72	51.5	5.48
8	0.96	51.93	5.91
10	1.2	52.16	6.14
12	1.44	52.32	6.3
18	2.16	52.61	6.59
24	2.88	52.78	6.76
30	3.6	52.89	6.87

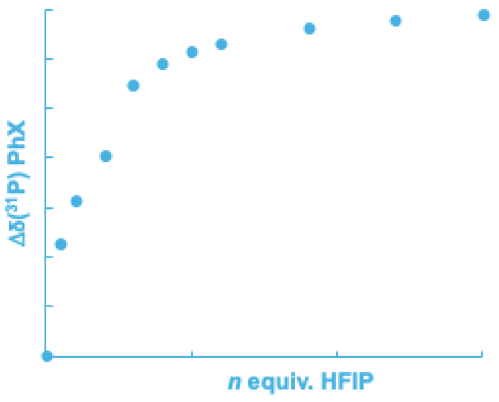

7. References

[37] B. Picard, B. Gouilleux, T. Lebleu, J. Maddaluno, I. Chataigner, M. Penhoat, F.-X. Felpin, P. Giraudeau, J. Legros, *Angew. Chem. Int. Ed.* **2017**, *56*, 7568–7572.

[42] P.-Y. Renard, H. Schwebel, P. Vayron, L. Josien, A. Valleix, C. Mioskowski, *Chem. Eur. J.* **2002**, *8*, 2910–2916.

[46] A. B. Smith, L. Ducry, R. M. Corbett, R. Hirschmann, *Org. Lett.* **2000**, *2*, 3887–3890.

[47] S. Mansour, A. Delaune, M. Manneveau, B. Picard, A. Claudel, C. Vallières, L. Sigot, P.-Y. Renard, J. Legros, *Green Chem.* **2021**, *23*, 7522–7527.
